# Targeting eIF4F translation initiation complex with SBI-756 sensitises B lymphoma cells to venetoclax

**DOI:** 10.1038/s41416-020-01205-9

**Published:** 2020-12-14

**Authors:** Lee-or Herzog, Beth Walters, Roberta Buono, J. Scott Lee, Sharmila Mallya, Amos Fung, Honyin Chiu, Nancy Nguyen, Boyang Li, Anthony B. Pinkerton, Michael R. Jackson, Robert J. Schneider, Ze’ev A. Ronai, David A. Fruman

**Affiliations:** 1 Department of Molecular Biology & Biochemistry, University of California, Irvine, CA 92697 USA; 2 New York University School of Medicine, New York, NY USA; 3 Sanford Burnham Prebys Medical Discovery Institute, La Jolla, CA 92037 USA; 4 Genomics Institute of the Novartis Research Foundation, San Diego, CA 92121 USA; 5 Benaroya Research Institute, Seattle, WA 98101 USA

**Keywords:** B-cell lymphoma, Molecular medicine

## Abstract

**Background:**

The BCL2 inhibitor venetoclax has shown efficacy in several hematologic malignancies, with the greatest response rates in indolent blood cancers such as chronic lymphocytic leukaemia. There is a lower response rate to venetoclax monotherapy in diffuse large B-cell lymphoma (DLBCL).

**Methods:**

We tested inhibitors of cap-dependent mRNA translation for the ability to sensitise DLBCL and mantle cell lymphoma (MCL) cells to apoptosis by venetoclax. We compared the mTOR kinase inhibitor (TOR-KI) MLN0128 with SBI-756, a compound targeting eukaryotic translation initiation factor 4G1 (eIF4G1), a scaffolding protein in the eIF4F complex.

**Results:**

Treatment of DLBCL and MCL cells with SBI-756 synergised with venetoclax to induce apoptosis in vitro, and enhanced venetoclax efficacy in vivo. SBI-756 prevented eIF4E-eIF4G1 association and cap-dependent translation without affecting mTOR substrate phosphorylation. In TOR-KI-resistant DLBCL cells lacking eIF4E binding protein-1, SBI-756 still sensitised to venetoclax. SBI-756 selectively reduced translation of mRNAs encoding ribosomal proteins and translation factors, leading to a reduction in protein synthesis rates in sensitive cells. When normal lymphocytes were treated with SBI-756, only B cells had reduced viability, and this correlated with reduced protein synthesis.

**Conclusions:**

Our data highlight a novel combination for treatment of aggressive lymphomas, and establishes its efficacy and selectivity using preclinical models.

## Background

The mammalian target of rapamycin (mTOR) is a central regulator of cell growth and proliferation, as well as a target for therapeutics in cancer and other diseases.^1^ The two complexes that facilitate signal transduction in the mTOR pathway are mTORC1 and mTORC2. mTOR-activating mutations occur in diffuse large B cell lymphoma (DLBCL),^2^ and elevated mTORC1 activity correlates with chemotherapy resistance and poor prognosis in pre-B acute lymphoblastic leukaemia (B-ALL).^3^

mTORC1 substrates include S6 kinases (S6Ks) and eukaryotic translation initiation factor 4E (eIF4E)-binding proteins (4E-BPs). Phosphorylation of 4E-BP releases its inhibition of eIF4E. Upon release, eIF4E binds the scaffolding protein eIF4G1 and the RNA helicase, eIF4A, to form the eIF4F protein translation initiation complex that binds to the 5’ cap of certain mRNAs and facilitates cap-dependent translation.^4–6^ Phosphorylation of 4E-BP1 correlates with high risk in B-ALL and chronic lymphocytic leukaemia,^3,7^ while mTOR inactivation impairs B-ALL survival.^8,9^

Several mTORC1 inhibitors have been developed and investigated in the treatment of B-cell non-Hodgkin’s lymphoma (NHL), yet each has caveats. Rapamycin and its analogues (rapalogs) are only partial inhibitors of mTORC1 that do not effectively suppress 4E-BP1 phosphorylation.^10^ Second generation mTOR kinase inhibitors (TOR-KI) act as ATP-competitive inhibitors and fully inhibit both mTORC1 and mTORC2.^11,12^ One candidate TOR-KI studied in our lab, MLN0128/TAK-228,^13^ has entered a phase 2 clinical trial in B-ALL. TOR-KI have shown improved proapoptotic activity in preclinical studies^11,12^ yet their therapeutic potential is limited by several factors including toxicity,^14^ adaptive survival signalling^15^ and mTOR resistance mutations.^16^

A promising alternative is to identify and target processes downstream of mTOR that are selectively required for cancer cell survival. One such process is cap-dependent mRNA translation controlled by the eIF4F complex.^4–6^ Compared to normal cells, cancer cells are “addicted” to high levels of eIF4F activity,^4–6^ as demonstrated using eIF4E heterozygous mice, which were healthy yet resistant to Ras-driven tumorigenesis.^17^ Various malignant cells rely on cap-dependent translation of specific mRNAs encoding many oncogenes, cell cycle regulators and prosurvival factors^4–6^ including BCL2 family members (e.g. MCL-1^18^). Hence, targeting cap-dependent translation downstream of mTOR could enhance efficacy of other proapoptotic therapies.

Currently available eIF4F inhibitors have liabilities, including low potency, lack of selectivity or poor pharmacological properties.^5^ Here we use the novel small molecule inhibitor of eIF4G1, named SBI-756, to target the eIF4F translation initiation complex.^19^ SBI-756 is a small molecule that binds to eIF4G1 and prevents its interaction with eIF4E.^19^ In our previous study, SBI-756 inhibited melanoma in vitro and in vivo.^19^

Venetoclax is a small molecule BH3 mimetic drug that selectively binds BCL2 and inhibits its prosurvival function.^20,21^ Since initial FDA approval of venetoclax for treatment of chronic lymphocytic leukaemia (CLL) patients with 17p chromosomal deletion,^22^ additional combination regimens have been approved.^23,24^ Novel combinations are needed to improve responses in NHL, where single agent venetoclax has limited activity.^25^ Here we tested the efficacy of SBI-0640756 (SBI-756 hereafter) in NHL cell lines, alone and in combination with venetoclax. We find that SBI-756 synergises with venetoclax in DLBCL and mantle cell lymphoma (MCL) cells in vitro, and promotes tumour regression in vivo. SBI-756 at nanomolar concentrations disrupts the eIF4G1:eIF4E interaction in cells, reprogramming mRNA translation and sensitising to apoptosis. Lymphoma cells with natural or engineered loss of 4E-BP1 were resistant to TOR-KIs yet retained sensitivity to SBI-756. Mechanistic experiments showed that SBI-756 had a selective effect on translation efficiency of components of the translation machinery, leading to reduced protein synthesis rates. These results identify disruption of eIF4F assembly as a promising approach to enhance venetoclax efficacy in NHL.

## Methods

### Duolink proximity ligation assay (PLA)

We performed PLA as described before.^26^ Briefly: 2×10e6 cells were treated for 4 h as indicated. Cells were washed with 1× phosphate buffered saline (PBS) (Corning, NY) and fixed with 4% paraformaldehyde (Thermo Fisher Scientific). CometSlides (Trevigen, Gaithersburg, MD) were coated with Poly-L-Lysine 0.1% solution (Sigma–Aldrich (SA), St. Louis, MO), and cells were allowed to adhere. We followed the protocol of Duolink PLA;^27^ briefly: Cells were blocked using Duolink blocking solution, followed by probing with primary antibodies for eIF4G1 (Cell signaling Technologies, Danvers, MA, Cat. #2858, 1:200 dilution) and eIF4E (BD Biosciences, San Diego, CA, Cat. #610269, 2.5 µg/ml final). Next, cells were incubated with Duolink In Situ PLA Probe Anti-Rabbit PLUS (Cat. # DUO92002) and Duolink In Situ PLA Probe Anti-Mouse (Cat. # DUO92004) and allowed to ligate using ligation mix. Next, amplification and washes were performed as instructed and the slides were mounted using media containing DAPI. Slides were imaged using Leica TCS SP8 confocal microscope. Signal obtained was quantified using ImageJ software, and normalised to the number of cells per field (using DAPI nuclei staining). Images shown indicate the signal (Orange Duolink^TM^) and nuclei for each field imaged, while graphs presented indicate ratio values of signal per cell in each field imaged.

### Mice strain and compounds administration in vivo

Thirty-two NOD scid gamma (NSG) healthy immunodeficient mice (Jackson Laboratories, Bar Harbor, ME) were used for in vivo experiments (8 weeks old, 23 gram in average) after 7 days acclimation in animal facility. Animal studies were approved by the Institutional Animal Care and Use Committee at UC Irvine. Female NSG mice were injected subcutaneously (s.c.) with 1 × 10e6 OCI-LY1 cells/mouse. We anesthetised the mice (100 mg/kg ketamine—10 mg/kg xylazine) and cells were injected in total volume of 200 µl along with Matrigel (Corning) for providing a supportive environment for tumour development. Once tumour size reached 110 mm^3^ volume, mice were randomised into treatment groups (*n* = 8) and treated daily (non-blinded way) for 5 days. Each mouse body weight was examined throughout the trial to identify potential toxicity or changes in dosing parameters. Also, tumour sizes were monitored daily and recorded. All mice were monitored for clinical signs of pain or distress during the procedures and daily during tumour measurements; no clinical signs were observed. At the end of five days of dosing, each mouse was weighed, sacrificed (according to IACUC guidelines, using CO_2_ inhalation followed by cervical dislocation) and tumours were excised for analysis. Analysis of tumours included tumour size, weight and preparation of single-cell suspension without exclusion of data points. Cells extracted were fixed using 4% PFA and used for intracellular staining as well as PLA.

### Polysome profiling

Cells were grown to ~70% confluence. Cycloheximide (0.1 mg/mL final concentration) was added to the medium for 5 min at 37 °C to arrest the ribosomes. The cells were washed twice with PBS containing 0.1 mg/mL cycloheximide, and then pelleted. The supernatant was removed, and the pellet was flash frozen. Cell pellets were lysed for 10 min on ice with 400 µL polysome extraction buffer (15 mM Tris-Cl, pH7.4, 15 mM MgCl_2_, 0.3 M NaCl, 0.1 mg/mL cycloheximide, 0.1 mg/mL heparin, 1% Triton X-100). The lysates were cleared by centrifugation at 13,200 × *g* for 10 min. Equal RNA concentrations were layered onto 20–50% sucrose gradients. Gradients were sedimented at 151,263 × *g* for 103 min in a SW55 Ti rotor at 4 °C. An ISCO UA-6 (Teledyne, Thousand Oaks, CA) fraction collection system was used to collect 12 fractions, which were immediately mixed with 1 volume of 8 M guanidine HCl. RNA was precipitated from polysome fractions by ethanol precipitation and dissolved in 20 µL of H_2_O. Briefly, fractions were vortexed for 20 s. 600 µL of 100% ethanol was added, and fraction was vortexed again. Fractions were incubated overnight at −20 °C to allow for complete RNA precipitation. Fractions were centrifuged at 13,200 rpm for 30 min at 4 °C. The RNA pellet was washed with 75% ethanol. The pellet was resuspended in 400 µL 1× Tris-EDTA (pH 8.0). 0.1 volumes of 3 M NaOAc (pH 5.3) and 2.5 volumes 100% ethanol were added, and fractions were incubated at −20 °C to precipitate RNA. Fractions were centrifuged at 13,200 rpm for 30 min at 4 °C. The RNA pellet was washed with 75% ethanol. RNA was resuspended in 20 µL H_2_O. Total RNA samples were isolated from cell lysates using Trizol per the manufacturer’s instructions.

### RNA-seq and analysis

Fractions containing four or more ribosomes (considered well-translated) were pooled and RNA quality was measured by a Bioanalyzer (Agilent Technologies). RNA-seq was carried out by the New York University School of Medicine Genome Technology Core using the Illumina HiSeq 4000 single read. To examine differences in transcription and translation, total mRNA and polysome mRNA were quantile-normalised separately. Statistical analysis was performed using RIVET.^28^ GO analysis was performed using the DAVID online tool.

### Statistical analysis

The number “n” of biological replicates for each experiment is indicated in the figure legends. Two-way ANOVA for multiple comparisons was performed where indicated while considering sample independence, variance equality and normality. ANOVA analysis while adjusting for multiple comparisons was performed for the in vivo experiment described to test for tumour growth. Student *t*-tests were applied to population means assuming equal variance (standard deviations within two-fold). The use of one- versus two-sample tests, and paired versus unpaired comparisons, was justified by the experimental design as indicated in the Figure Legends.

Additional standard and published methods are provided in the Supplementary Materials.

## Results

### Constitutively active 4E-BP1 mutant sensitises DLBCL cells to venetoclax, similar to TOR-KI treatment

mTOR inhibitors enhance killing of DLBCL cells by BH3 mimetics, such as venetoclax (ABT-199), ABT-263 or ABT-737.^29^ To evaluate the role of the 4E-BP/eIF4E axis in this sensitisation, we used a doxycycline (DOX)-inducible system to express wild-type 4E-BP1 or a constitutively active form in which all five serine/threonine phosphorylation sites were changed into alanine (5A mutant). Since 4E-BP1-5A cannot be phosphorylated by mTORC1, expression of this mutant prevents eIF4E from associating with eIF4G1 and other proteins to form the eIF4F complex.^30^ We generated OCI-LY1 DLBCL cells expressing the reverse tetracycline transactivator (rtTA) protein and either empty vector (EV), WT 4E-BP1 or 4E-BP1 mutant (5A). Addition of DOX induced expression of the mutant protein after 16 h (Fig. S1a). Next, we treated cells (±DOX) for 48 h with a range of venetoclax concentrations in combination with either vehicle (DMSO), or TOR-KI (MLN0128 100 nM). As expected, TOR-KI treatment sensitised to venetoclax as shown by reduced IC50 values (Fig. S1b). Notably, OCI-LY1 cells expressing the active 4E-BP1 (5A) and treated with vehicle (lane 5, Fig. S1a) were as sensitive to venetoclax as control cells (WT or EV) treated with TOR-KI (lanes 2 and 4 Fig. S1b). TOR-KI further increased sensitisation to venetoclax in cells expressing 4E-BP1 MUT. A similar sensitisation was observed in OCI-LY1 cells expressing 4E-BP1 5A and treated with navitoclax (ABT-263)—an inhibitor of BCL2, BCL2L1 (BCL-xL), and BCL-W (Fig. S1c, d). In summary, the ability of the 4E-BP1 mutant to phenocopy the effect of TOR-KI demonstrates that targeting the 4E-BP1/eIF4E arm of mTORC1 signalling is a promising approach for sensitisation of DLBCL cells to venetoclax treatment.

### SBI-756 prevents eIF4E:eIF4G1 association and reduces cap-dependent translation in lymphoma cells

**Fig. 1:**
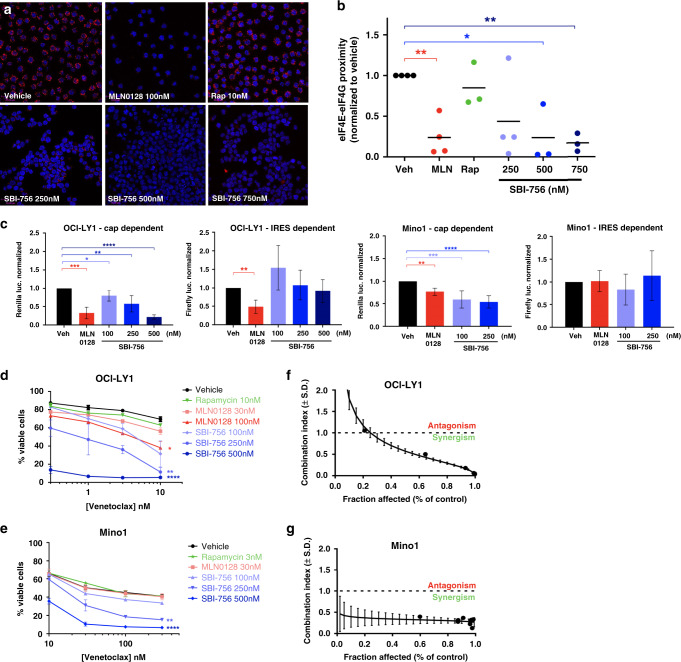
SBI-756 prevents eIF4E-eIF4G interaction and cap-dependent translation. **a** Cells were tested for eIF4E:eIF4G1 association via Proximity Ligation Assay (PLA). OCI-LY1 DLBCL cells were treated for 4 h with either vehicle (DMSO), MLN0128 100 nM, Rapamycin 10 nM or increasing concentrations of SBI-756 (250–750 nM). Scale bar = 33 µm. Representative images of at least three fields are shown. **b** Quantification of eIF4E:eIF4G1 interaction for each treatment. The signal indicating eIF4E:eIF4G interaction was measured from the entire field for each treatment (single channels acquired) and was normalised to the number of cells imaged (DAPI staining indicating cells/image). Relative ratios are graphed. *p < 0.05; **p < 0.01. *n* = 3 or 4, as indicated. Paired one-sample *t*-test. **c** OCI-LY1 or Mino1 cells were electroporated to introduce the dual-luciferase reporter in which 5’ cap-dependent untranslated region (5’-UTR) was conjoined to a *Renilla* (*Renilla reniformis)* luciferase reporter, while the Internal Ribosomal Entry Site (IRES) was conjoined to a firefly (*Photinus pyralis*) luciferase reporter. Cells were treated for 16 h with vehicle (DMSO), MLN0128 100 nM, or increasing concentrations of SBI-756 (250–750 nM). Both *Renilla* luciferase (cap-dependent translation) and firefly luciferase (IRES dependent translation) signals were measured and each was normalized to vehicle contol. *p < 0.05, **p < 0.01, ***p < 0.005. One-sample *t*-test vs. DMSO control. *n* = 3. Viability of **d** OCI-LY1 and **e** Mino1 cells treated for 48 h with increasing venetoclax concentrations in combination with vehicle (DMSO) control or various inhibitors as indicated. Viability was assessed using Annexin V and PI staining. *p < 0.05; **p < 0.01; ****p < 0.001. We performed independent *t*-tests (unpaired *t*-tests) and compared each treatment group to vehicle treated group. We also performed an adjustment for multiple comparisons for each *t*-test performed. We calculated IC50 values for each cell line tested based of the viability assays performed: **f** OCI-LY1 IC50s–venetoclax 23.6 nM, SBI-756 209.7 nM; **g** Mino1 IC50s–venetoclax 749.9 nM, SBI-756 340.3 nM. Isobologram plots were graphed based on *Chou-Talala*y method for synergy calculation (combination index)^11^ using median effect method for cell lines treated for 48 h with combinations of SBI-756 and venetoclax at fixed ratios.

Next, we took a chemical approach to disrupting eIF4F. Previously, we showed that the cell-permeable compound SBI-756 binds to eIF4G1 and disrupts formation of the mRNA cap-binding complex in melanoma cells and in fibroblasts.^19^ To assess the effect of SBI-756 on eIF4F formation in lymphoma cells, we used a proximity ligation assay (PLA) to quantitate the interaction of eIF4E and eIF4G1 in situ. As expected, treatment of OCI-LY1 cells with the TOR-KI compound MLN0128 suppressed eIF4E:eIF4G1 association whereas rapamycin, a weak inhibitor of 4E-BP1 phosphorylation,^10,30^ had no significant effect (Fig. 1a, b). SBI-756 reduced eIF4E:eIF4G1 interaction in a dose-dependent manner (Fig. 1a, b). Similar results were observed in OCI-LY8 cells (Fig. S2a, b). Quantification of eIF4E:eIF4G1 interaction indicated a significant reduction by 500 nM SBI-756 in OCI-LY1 (76%, Fig. 1b) and 250 nM in OCI-LY8 (83%, Fig. S2b).

We used dual-luciferase reporter assays to test the ability of TOR-KI and SBI-756 to reduce cap-dependent and IRES-dependent translation. Following 16 h of treatment of OCI-LY1 and the MCL cell line Mino1, SBI-756 in the range of 100–500 nM selectively reduced cap-dependent luciferase expression (Fig. 1c). Similar results were observed in two additional DLBCL lines (OCI-LY8, SU-DHL6) and in the Maver1 MCL line (Fig. S2c). Likewise, MLN0128 significantly reduced accumulation of cap-dependent luciferase (Figs. 1c and S2).

### SBI-756 does not change mTOR substrate phosphorylation

A potential advantage of selective eIF4F targeting is that this approach should preserve activity of mTOR, reducing on-target toxicities associated with mTOR inhibition. Compared to the TOR-KI compound Torin-1, SBI-756 (1 µM or lower concentration) did not inhibit phosphorylation of mTORC1 or mTORC2 substrates in melanoma cells.^19^ To test whether SBI-756 alters mTOR activity in lymphoma cells, we measured mTORC1 and mTORC2 substrate phosphorylation. As expected, MLN0128 significantly reduced phosphorylation of both mTORC1 (p-S6, p-4E-BP1) and mTORC2 (AKT) substrates, whereas rapamycin reduced only p-S6 (Fig. S3a, b). In contrast, treatment with SBI-756 did not alter phosphorylation of any mTOR substrates tested, indicating that the mTOR signalling pathway was not altered by SBI-756 treatment (Fig. S3a, b). Similar results were obtained in other DLBCL cell lines (Fig. S3c, d). These results support the conclusion that SBI-756 directly disrupts the eIF4F complex without altering activity of mTORC1 or mTORC2.

### SBI-756 synergises with venetoclax to increase apoptosis

To test the ability of SBI-756 to promote apoptosis and sensitise lymphoma cells to venetoclax, we evaluated seven GCB-DLBCL and five MCL cell lines (Figs. 1d, e and S4). We treated the cells with titrated concentrations of venetoclax as a single agent, or in combination with either MLN0128 or SBI-756 (Figs. 1d, e and S4a, b) and performed viability assays. Eight of the cell lines tested had reduced viability following 48 h treatment with venetoclax as a single agent. Ten cell lines showed reduced viability following SBI-756 treatment as a single agent. In five of the SBI-756-sensitive DLBCL lines (OCI-LY1, OCI-LY8, OCI-LY18, SU-DHL-4, SU-DHL-6) and four of the MCL lines (Mino, Jeko1, MAVER-1, CCMCL-1), the combination of SBI-756 and venetoclax caused more cell death than individual agents (Figs. 1d, e and S4). In comparison, MLN0128 only sensitised to venetoclax in one cell line (OCI-LY1) (Fig. 1d) despite effectively suppressing mTOR signalling outputs in other cell lines such as OCI-LY8 (Fig. S3a, c). Supporting an apoptotic mechanism, the combination of SBI-756 with venetoclax induced caspase-dependent death, as demonstrated by rescue of viability in cells co-treated with the pan-caspase inhibitor QVD-OPH (Fig. S5a). Moreover, venetoclax treatment with or without SBI-756 led to cleavage of caspase-3 and PARP (Fig. S5b). Treatment with SBI-756 alone did not induce cleavage of caspase-3 or PARP (Fig. S5b).

Next, we chose sensitive DLBCL and MCL cells to further evaluate synergy between venetoclax and SBI-756 (Figs. 1f, g and S4c). We measured viability in cells treated for 48 h with fixed ratios of venetoclax and SBI-756 and assessed synergy using the Chou-Talalay method.^31^ Indeed, SBI-756 synergised with venetoclax (combination index < 1) in both OCI-LY1 (DLBCL) and Mino1 (MCL) cells (Fig. 1f, g). Furthermore, SBI-756 was found to synergise with venetoclax in five more cell lines tested (Fig. S4c).

SBI-756 did not reduce viability or sensitise to venetoclax in the OCI-LY7 DLBCL line and had minimal effect in a subline of OCI-LY1 cells that we selected for resistance to SBI-756 (Fig. S4a). In both these cell lines, SBI-756 treatment did not prevent the eIF4E:eIF4G1 interaction measured by PLA (Fig. S4d).

### Lymphoma cells lacking 4E-BP1 are resistant to TOR-KI yet remain sensitive to SBI-756

**Fig. 2:**
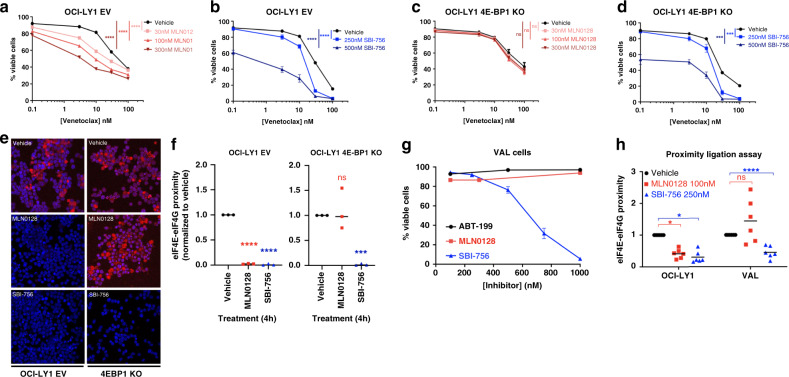
Lymphoma cell lacking 4E-BP1 are resistant to TOR-KI yet are sensitive to SBI-756 treatment. OCI-LY1 cells stably expressing Cas9 were transfected with guide RNAs. Clones were isolated that carried an empty vector (**a**, **b**, **e**); or sgRNAs specific for 4E-BP1 (*4E-BP1 KO*) (**c**, **d**, **f**). Parental OCI-LY1 cells gave comparable results to EV transfected OCI-LY1 (data not shown). **a**–**d**. Cells were treated for 48 h with titrated amounts of venetoclax without (vehicle) or with MLN0128 (**a**, **c**) or SBI-756 (**b**, **d**). Viability was assessed using annexin V and PI staining. *n* = 4. *p < 0.05, ***p < 0.01, ****p < 0.005, ns not significant. Statistics were done using Two-way ANOVA. **e**, **f** PLA comparing parental OCI-LY1 to 4E-BP1 KO cells after 4 hours treatment. Five fields were imaged and quantified by a blinded observer (**e**). Data are plotted for individual experiments with the means of each group indicated by a horizontal line (**f**). *n* = 3. *p < 0.05, **p < 0.01, ***p < 0.005, ****p < 0.001, ns not significant. Paired one-sample *t*-test vs. control. **g** Viability assay of VAL cells (naturally lacking 4E-BP1^6^) following 48 h of treatment with venetoclax, MLN0128 or SBI-756 (all within 100–1000 nM range). **h** PLA comparing OCI-LY1 to VAL cells (naturally lack 4E-BP1^6^) following 4 h of treatment. Fields imaged and quantified by a blinded observer. Data are plotted for individual experiments with the means of each group indicated by a horizontal line. *n* = 3. *p < 0.05, **p < 0.01, ***p < 0.005, ****p < 0.001, ns not significant. Paired one-sample *t*-test vs. control.

Many cancer cells exhibit a reduction of 4E-BP expression^32,33^ or increase in eIF4E expression,^34,35^ enabling cap-dependent translation even following mTORC1 inhibition. Indeed, the eIF4E/4E-BP ratio can predict efficacy of mTOR targeted therapies.^36^ Previously we reported that SBI-756 can fully suppress proliferation in 4E-BP1/4E-BP2 double knockout fibroblasts that are partially resistant to the TOR-KI compound Torin-1.^19^ To determine whether SBI-756-induced DLBCL death is 4E-BP-dependent, we used CRISPR/Cas9 genome editing to generate clones of OCI-LY1 cells lacking 4E-BP1 (Fig. S6). We compared these 4E-BP1 knockout (KO) clones to OCI-LY1 cells expressing Cas9 and empty sgRNA vector (EV) in viability assays, using a range of venetoclax concentrations without or with MLN0128 or SBI-756. As in parental cells, 10 nM venetoclax caused ~10% death in the EV and 4E-BP1 KO lines (Fig. 2a–d); higher concentrations of venetoclax caused more death. MLN0128 and SBI-756 sensitised to venetoclax in EV cells (Figs. 2a, b) as in parental OCI-LY1 (Fig. 1d). Notably, OCI-LY1 cells lacking 4E-BP1 were completely resistant to MLN0128 yet remained sensitive to SBI-756 alone or in combination with venetoclax (Figs. 2c, d and S6). Similarly, SBI-756 reduced eIF4E:eIF4G1 interaction among all cells, whereas treatment with MLN0128 reduced interaction only in control cells containing 4E-BP1 (Figs. 2e, f). Our findings indicate that in cells lacking 4E-BP1 expression, SBI-756 retains its effect and prevents eIF4F formation, thus sensitising those cells to BCL2 inhibition.

To further support this conclusion, we used VAL cells, a DLBCL line lacking 4E-BP1^32^ (Fig. S3d). Consistent with previous observations,^32^ MLN0128 at concentrations up to 3 µM did not affect VAL cell viability (Fig. 2g) or eIF4E:eIF4G interaction (Fig. 2h). In contrast, SBI-756 reduced viability of VAL cells (Fig. 2g) and disrupted the eIF4E:eIF4G interaction (Fig. 2h). VAL cells were completely insensitive to venetoclax, with or without SBI-756. Nevertheless, these data confirm that prevention of eIF4F complex formation is achievable using SBI-756, even among cells lacking 4E-BP1 (thus insensitive to TOR-KI).

### SBI-756 is effective and well tolerated in vivo

**Fig. 3:**
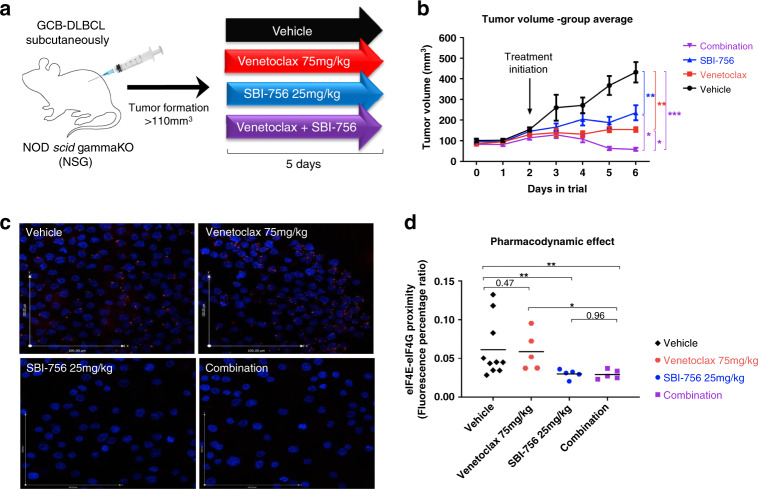
SBI-756 sensitises to venetoclax treatment in vivo. **a**. NOD-scid-IL2-Rgamma-/- (NSG) immunodeficient mice were injected with 1x10e7 OCI-LY1 cells s.c. (7-8 mice per group). Once palpable tumours were established (>110 mm^3^), mice were treated with 0.1% DMSO vehicle (i.p. and p.o.), SBI-756 25 mg/kg (i.p.), venetoclax 75 mg/kg (p.o.) or combination (i.p. and p.o.) for five consecutive days (Fig. 3). The tumour weight, single-cell extractions and pharmacodynamics were monitored on the day of sacrifice, while mouse body weight and tumour volume were monitored throughout the trial. **b**Tumour volume was calculated each day using the formula: v = 4/3πr_1_r_2_r_3_ and presented as mean ± SEM. *p < 0.05, **p < 0.01, ***P < 0.005, ****p < 0.001, ANOVA, *p* value has been adjusted for multiple comparisons (using Tukey’s adjustment). **c** eIF4E:eIF4G1 association measured by PLA assay on single cells isolated from tumours. SBI-756 treatment (alone/combination) reduced association of eIF4E:eIF4G1. scale bar = 100 µm. **d** Quantification of eIF4E:eIF4G1 interaction for each treatment group. Each point represents one mouse in the group that was treated for 5 consecutive days and euthanised 4 h after the last dose. The signal indicating eIF4E:eIF4G interaction was measured from the entire field for each treatment (single channels acquired) and was normalised to the number of cells imaged (DAPI staining indicating cells/image). Relative ratios are graphed. Data are plotted for individual experiments with the means of each group indicated by a horizontal line. *p < 0.05; **p < 0.01, ANOVA, *p* value adjusted for multiple comparisons.

We assessed whether sensitisation of DLBCL to venetoclax treatment by cotargeting eIF4F could be recapitulated in vivo. We injected NSG mice with OCI-LY1 (s.c.) and once palpable tumours were established, treated with vehicle, venetoclax, SBI-756 or their combination for 5 consecutive days (Fig. 3a). There was no significant change in body weight among the different groups (Fig. S7a), indicating that the treatments were well tolerated. Both venetoclax and SBI-756 significantly slowed tumour growth when administered as single agents (Figs. 3b, S7b, c). Notably, venetoclax and SBI-756 combination caused tumour regression and significantly reduced tumour volume (7/7 mice) when compared to SBI-756 (1/8 mice) or venetoclax (2/8 mice) as single agents or vehicle alone (0/8 mice). Together these results indicate that a synergistic relationship between venetoclax and SBI-756 occurs not only in vitro but also in vivo.

### SBI-756 has a pharmacodynamic effect in vivo

To assess whether SBI-756 treatment prevents eIF4E:eIF4G1 interaction in vivo, lymphoma tumours excised from euthanised mice were dissociated into single cells and subjected to PLA. Analysis of the samples obtained showed a reduction in eIF4E:eIF4G1 interaction among tumours treated with SBI-756 as a single agent or in combination with venetoclax, compared to vehicle or venetoclax alone (Fig. 3c, d). Additionally, we performed intracellular staining to measure phosphorylation levels of mTOR substrates, S6 kinase and 4E-BP1, among the samples extracted from the tumours to test for alteration in mTOR kinase activity. No significant changes were observed in mTOR substrate phosphorylation in mice treated with SBI-756 and/or venetoclax in vivo (Fig. S7d, e). These results suggest that SBI-756 potentiates venetoclax efficacy in vivo by preventing eIF4E:eIF4G1 interaction without affecting mTOR activity.

### SBI-756 has both direct and indirect effects on mRNA translation

Chemical inhibition of mTORC1 or eIF4F in cancer cells selectively suppresses translation of mRNAs with specific features in the 5′ untranslated region (UTR).^37^ These eIF4F-sensitive mRNAs include several that encode prosurvival proteins, including MCL-1, BCL-xL and survivin.^18^ To determine whether SBI-756 affects expression of these factors in DLBCL cells, we measured expression of candidate proteins by western blot. In cells growing asynchronously, 4-h treatment with MLN0128 or SBI-756 did not change expression of these candidate proteins (data not shown). In a previous study we found that serum starvation of DLBCL cells, followed by re-addition of serum without or with mTOR inhibitors, revealed consistent changes in protein expression.^32^ Taking this approach, we observed modest and variably reduced expression (~2-fold) of MCL-1, BCL-xL and survivin among MLN0128 and SBI-756 treated cells (Fig. S8a, b). Expression of eIF4E or eIF4G1 were also reduced following SBI-756 or MLN0128 treatment (Fig. S8a, b). For each of these targets (MCL-1, BCL-XL, survivin, eIF4E or eIF4G1), the abundance of mRNA was not significantly changed in cells treated with SBI-756 or MLN0128 (with the exception of survivin transcripts elevated in cells treated with 500 nM SBI-756) (Fig. S8c).

**Fig. 4:**
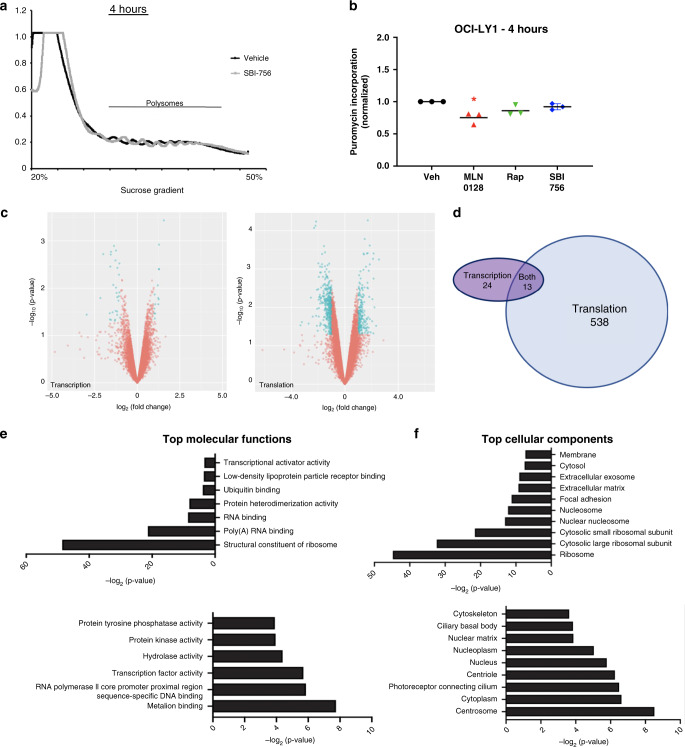
Selective reduction of mRNA translation following 4 h SBI-756 treatment. **a** Polysome traces of OCI-LY1 cells treated with SBI-756 or without (vehicle) for 4 h. Results are representative of three independent samples. **b** Quantification of puromycin incorporation in OCI-LY1 cells treated with vehicle, MLN0128 (30 nM), rapamycin (10 nM) or SBI-756 250 nM for 4 h with addition of puromycin to the media for the last 20 min. **c** Genome-wide transcription and translation mRNA profiling of 25 million cells with or without SBI-756 treatment. Results are from two independent studies. Total mRNA and purified fractions containing four or more bound ribosomes were sequenced using Illumina HiSeq 4 single read. Volcano plots represent differences in transcription (left) and translation (right). Transcription and translation parameters were *p* ≤ 0.05 and −1.0 ≤ log2 ≥ 1.0. Blue dots identify mRNAs significantly changed in abundance, and red dots identify mRNAs not significantly changed. Statistical analysis was performed using the limma R package via RIVET (Ernlund AW et al., BMC Genomics 2018). **d** Venn diagram comparing significant differences in transcription and translation. **e** The top molecular functions of mRNAs significantly down (top) or upregulated (bottom) in translation. **f** The top cellular components of mRNAs significantly down (top) or upregulated (bottom) in translation.

To gain a broad, unbiased view of how SBI-756 affects mRNA translation efficiency, we treated OCI-LY1 cells with vehicle (DMSO) or SBI-756 (250 nM) for 4 h, and isolated RNA from heavy and light polysome fractions, as well as total cellular RNA. The 4-h treatment did not cause detectable changes in RNA distribution from heavy to light polysomes (Fig. 4a) and did not change rates of overall protein synthesis (Fig. 4b). However, comparison of mRNAs associated with heavy polysomes to total mRNAs showed that SBI-756 selectively changed the translation efficiency of 538 mRNAs; 13 showed differences in both translation and transcription (Fig. 4c, d, Supp. Table 1). Only 24 genes showed changes in transcription alone (Fig. 4c, d), supporting the conclusion that SBI-756 is an on-target and selective inhibitor of mRNA translation.

Of the 538 mRNAs with selective change in translation, the majority (385) had reduced translation efficiency (Fig. 4c) (Supp. Table 1). Gene ontology (GO) analysis showed a highly significant enrichment for genes involved in mRNA translation. The most enriched biological processes for the downregulated genes (Fig. 4e top) include translation initiation, translation, rRNA processing and ribosomal small subunit assembly. Likewise, top molecular functions associated with the downregulated genes include structural constituent of the ribosome, RNA binding and poly(A) RNA binding (Fig. 4f). Among the translationally downregulated biological processes were negative regulators of apoptosis (Fig. 4e). This family contained 10 genes (Supplementary Table 2) yet did not include the candidates mentioned above (MCL-1, BCL-xL, survivin). Among the translationally upregulated mRNAs, only one was found to contain an IRES motif (*TP53*).

**Fig. 5:**
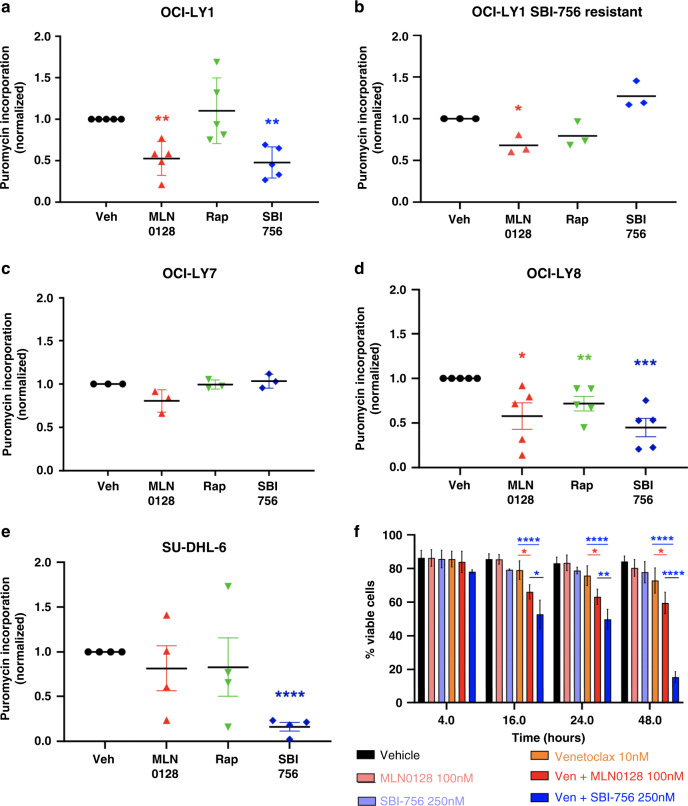
SBI-756 treatment reduces protein synthesis following 16 h of treatment. We treated cells with vehicle or inhibitors (MLN0128 (30 nM), rapamycin (10 nM), SBI-756 250 nM) and measured puromycin incorporation after 16 hr. **a** parental OCI-LY1 cells; **b** OCI-LY1 that were induced to resistance against SBI-756 (1 µM); **c** OCI-LY7; **d** OCI-LY8; **e** SU-DHL-6 cells. *p < 0.05, **p < 0.01. One-sample *t*-test vs. DMSO control. *n* = 3. **f** OCI-LY1 cells were treated with venetoclax 10 nM, MLN0128 100 nM, SBI-756 250 nM as single agents or in combination, as indicated. We have assessed their viability after 4, 16, 24 or 48 h of treatment using annexing V and PI staining and flow cytometry analysis. *p < 0.05, **p < 0.01. One-way ANOVA, compared to venetoclax as single agent.

The dramatic reduction in translation of mRNAs encoding ribosomal proteins and regulators at 4 h suggested that SBI-756 might indirectly reduce overall translation efficiency over time. Indeed, 16-h treatment with SBI-756 reduced rates of total protein synthesis in parental OCI-LY1 cells (Fig. 5a) but not in the SBI-756-resistant subline of OCI-LY1 cells (Fig. 5b). MLN0128 or SBI-756 treatment did not reduce protein synthesis in the OCI-LY7 cell line (Fig. 5c), in which these agents did not have cytotoxic activity (Fig. S4a). Nevertheless, SBI-756 did reduce protein synthesis in OCI-LY8 and SU-DHL-6 cell lines (Fig. 5d, e). This reduced protein synthesis could account for the sensitisation to venetoclax observed among these cells (Fig. S4a). Considering that SBI-756 reduced protein synthesis in OCI-LY1 cells at 16 h but not 4 h, we assessed cell survival at these timepoints as well as 24 h and 48 h. Sensitisation to venetoclax cytotoxicity was first evident 16 h following SBI-756 treatment (Fig. 5f), and was significantly greater than combination with TOR-KI.

### SBI-756 is not cytotoxic to human CD4+T cells, CD8+T cells, NK cells or monocytes

**Fig. 6:**
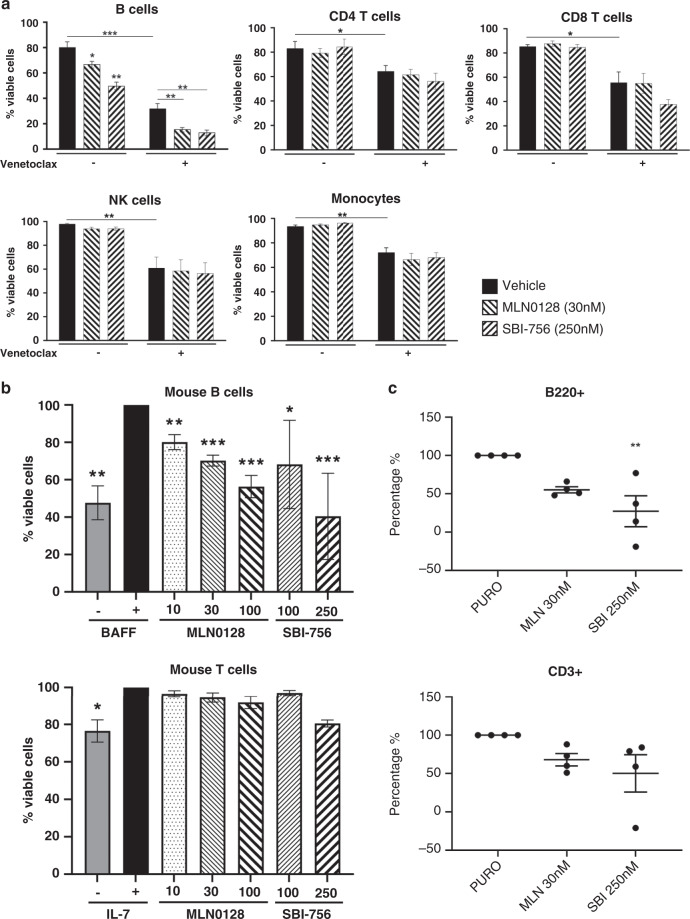
SBI-756 is not cytotoxic to human CD4 **+** T cells, CD8 **+** T cells, NK cells or monocytes. **a** Peripheral blood mononuclear cells (PBMCs) were obtained from healthy blood donors and were isolated as described previously.^38^ PBMCs (mean ± SD, *n* = 3) were cultured for 48 h with each agent MLN0128 (30 nM), SBI-756 (250 nM) alone or in combination with 100 nM venetoclax. Unpaired *t*-test vs. vehicle (DMSO) control. *n* = 3. *p < 0.05, **p < 0.01, ***p < 0.005 vs. DMSO control. Leukocyte subsets were distinguished by surface markers using flow cytometry. **b** Mouse splenocytes were isolated from C57BL/J mice. We performed B cell or T-cell purification using STEMCELL Mouse B cell or T-cell isolation kit, respectively. Next, we treated the cells as indicated for 48 h in the presence of cytokines (BAFF or IL-7), and their viability was measured using Annexin V and PI. *p < 0.05, **p < 0.01. One-sample *t*-test vs. control (control = B cells + BAFF; T cells + IL-7). *n* = 3. **c** Puromycin incorporation in mouse B cells (B220+) and T cell (CD3+) after 16 h of treatment with MLN0128 30 nM and SBI-756 250 nM. Data are showed as percentage of puromycin incorporation reduction vs vehicle. c vs. DMSO control. *n* = 4 **p < 0.01.

Lastly, we evaluated whether SBI-756 cytotoxic effects are selective for transformed lymphoma cells versus normal lymphocytes. We cultured PBMCs from healthy donors for 48 h with SBI-756 or MLN0128 alone, or in combination with venetoclax. Compared to vehicle treated cells, we observed a significant reduction in cell viability only among CD19 + B cells following MLN0128 or SBI-756 treatment (Fig. 6a). The effect of SBI-756 on B cell viability was dose dependent (Fig. S9a). Venetoclax alone greatly reduced viability of B cells, with partial effects on CD4 + and CD8 + T cells and natural killer cells as published before.^38^ However, there was no further effect when venetoclax was combined with either MLN0128 or SBI-756 (Fig. 6a). We also tested purified lymphocytes from mice, which are more readily available in quantities needed for correlation of functional and biochemical readouts. Similarly, MLN0128 and SBI-756 were selectively cytotoxic to mouse B cells but not mouse T cells cultured in supportive cytokines (Fig. 6b). The cytotoxic effect of SBI-756 in B cells at 48 h (Fig. 6b) correlated with suppression of protein synthesis selectively in B cells, measured after 16-h treatment (Fig. 6c).

## Discussion

Venetoclax is a BCL2-specific inhibitor whose use is expanding in hematologic malignancies.^20,21^ However, blood cancer cells frequently engage distinct mechanisms that can maintain survival following BCL2 inhibition. Therefore, responses to venetoclax are broader and more durable when the drug is combined with other agents that promote cell death through distinct mechanisms.^39^ In DLBCL, venetoclax combined with the standard of care (R-CHOP) is more effective than venetoclax monotherapy and components of the CHOP regimen can increase sensitivity to venetoclax in vitro.^40,41^ Incorporation of additional targeted agents has potential to further improve responses. Here, we report experiments showing great potential for sensitisation of NHL cells to venetoclax via combination with SBI-756, a potent inhibitor of cap-dependent translation that is active in cells in the 100–500 nM range. SBI-756 synergised with venetoclax to induce apoptosis in NHL cells while not interfering with mTOR signalling. SBI-756 prevented eIF4E:eIF4G1 interaction among sensitive cells (OCI-LY1 and OCI-LY8), but not among resistant cells (OCI-LY7 or OCI-LY1 induced to be resistant to SBI-756). After 4-h of treatment, SBI-756 also reduced translation efficiency of mRNAs encoding ribosomal proteins and rRNA processing factors. Consistent with reduced ribosome biogenesis, SBI-756 reduced overall protein synthesis after 16 h of treatment. Additionally, in DLBCL cell lines lacking 4E-BPs (naturally or genetically edited), SBI-756 retained ability to inhibit eIF4F formation and sensitise to venetoclax, whereas the TOR-KI compound MLN0128 lacked activity in this setting. SBI-756 synergy with venetoclax in vitro was recapitulated in vivo, with the combination reducing tumour progression that correlated with prevention of eIF4E-eIF4G1 interaction. Treatment with SBI-756 was tolerable in mice, and the compound selectively reduced survival of B cells in cultures of human PBMCs and murine lymphocytes. Together, these findings urge further investigation of eIF4F disruption for sensitisation to venetoclax and possibly other BH3 mimetics. In this regard, an initial experiment to sensitise DLBCL cell lines to an MCL-1 inhibitor (S63845) using SBI-756 (Fig. S9b) yielded a similar response as the venetoclax combination.

PI3K/mTOR signalling pathway activation has been correlated with poor prognosis and resistance to chemotherapy.^3^ Thus, this pathway remains an investigational target for cancer therapeutics, including rapalogs or TOR-KIs such as MLN0128/TAK-228.^11^ However, rapalogs have limited anticancer activity and the therapeutic window for TOR-KIs remains to be established. Targeting individual pathways downstream of mTOR might hold the key to developing better tolerated and more effective anticancer interventions. One process of particular interest is cap-dependent mRNA translation controlled by eIF4F.^4–6^ Genetic inhibition of eIF4F via inducible expression of the 4E-BP1 mutant sensitised to venetoclax in OCI-LY1 cells, an effect enhanced by the TOR-KI compound MLN0128 that reactivates endogenous 4E-BP1. Our studies of the eIF4G-binding compound SBI-756 in human lymphoma cell line models provide proof-of-concept for eIF4F disruption by a small molecule at sub-µM concentration. Further studies using pharmacologically optimised inhibitors of eIF4G1, or specific knockouts of eIF4G1 could clarify the importance of this component in lymphomagenesis or lymphocyte differentiation. In a distinct approach, rocaglate compounds targeting the eIF4A helicase can also enhance venetoclax cytotoxicity in lymphoma cells.^42^

In most of the DLBCL and MCL cell lines tested, 48-h treatment with SBI-756 alone had cytotoxic effects as measured by staining with Annexin V and propidium iodide. In OCI-LY1 and OCI-LY8 cells, this cytotoxic effect was prevented by co-incubation with a pan-caspase inhibitor. However, SBI-756 alone did not cause detectable cleavage of caspase-3 or PARP after 16 h, a time point where these markers were readily induced by venetoclax. It is possible that SBI-756 treatment alone triggers apoptosis at a later time point. Of note, two of the sensitive cell lines (OCI-LY1 and OCI-LY8) express mutant p53,^43^ suggesting that the mechanism of apoptosis is p53-independent.

Notably, the data suggest that SBI-756 is not cytotoxic to essential cellular mediators of immune function and immunotherapy efficacy (T cells and NK cells). A potential advantage for tolerability is that SBI-756 treatment (at concentrations of up to 500 nM) does not interfere with mTOR substrate phosphorylation while disrupting eIF4F complex assembly downstream of mTORC1. We did observe that higher concentrations of SBI-756 (>500 nM) do cause inhibition of the mTOR pathway; thus, future efforts should aim to optimise the selectivity of SBI-756 and similar agents. Another advantage of SBI-756 compared to TOR-KI treatment is its ability to suppress cap-dependent translation among cells lacking 4E-BP1, a finding relevant to tumours with reduced ratio of 4E-BPs to eIF4E. We used cells that naturally lack 4E-BP1.^32^ or were genome edited to lack 4E-BP1 to show that these cells remained sensitive to SBI-756 yet lacked TOR-KI sensitivity. Further experiments are needed to determine whether cells with high eIF4E or eIF4G1 retain sensitivity to SBI-756. These results suggest that B lineage cells have a unique dependence on the mTORC1/eIF4F axis for survival.

Polysome profiling showed that SBI-756 treatment in lymphoma cells alters translation efficiency of 538 genes, transcription of 24 genes, while 13 genes are regulated both transcriptionally and translationally. GO analysis of the translationally downregulated genes identified several groups related to mRNA translation, including structural constituent of the ribosome, ribosomal biogenesis, rRNA binding and translational elongation (Fig. S10). Together, those functions indicate two main effects of SBI-756 treatment: first, direct suppression of translation efficiency of a key subset of cellular mRNAs; second, indirect inhibition of the translation machinery that is needed to sustain protein synthesis. The two effects together (direct and indirect) reprogram mRNA translation in a way that promotes lymphoma cell death and sensitises to BCL2 inhibition. Supporting the correlation of protein synthesis inhibition and cell death, the OCI-LY7 cell line was resistant to SBI-756 cytotoxicity and did not show reduced protein synthesis rates. Moreover, protein synthesis was not reduced among OCI-LY1 cells selected for SBI-756 resistance. Interestingly, the selective cytotoxic effect of SBI-756 on B cells versus T cells correlated with suppression of global protein synthesis.

The polysome profiling dataset does not definitively establish acute effects of SBI-756 on translation efficiency of survival factors, such as ones reported previously to be eIF4F-dependent. Western blotting experiments suggested that SBI-756 reduces protein amounts of MCL-1 and survivin, whose expression is known to be sensitive to changes in cap-dependent translation.^44^ This reduction of prosurvival factors may block back-up mechanisms that the cells might use to adjust to BCL2 inhibition.^45^ Another possibility is that the broad reprogramming of translation produces subtle changes that alter the overall balance of prosurvival and proapoptotic proteins over time, tipping the balance towards cell death.^4,46^ Reducing ribosome production using inhibitors of ribosomal DNA transcription likewise promotes lymphoma cell death.^47^

The central role of mRNA translation in hallmarks of cancer has led to many distinct approaches to target regulatory components of the translation machinery.^4–6^ Natural products and synthetic small molecules have been identified with various mechanisms including: (i) interfering with eIF4E binding to the m^7^-GTP cap (for example, 4EGI-1^48^); (ii) inhibiting MNK kinases that phosphorylate eIF4E (CGP57380;^49^ eFT508^50^); (iii) disrupting the function of eIF4A helicase (Silvestrol^51^ and synthetic rocaglates^42,52^); (iv) inhibiting translational elongation (homoharringtonine^53^). Many of the compounds have low selectivity, weak potency or cell penetrance, poor pharmacological properties or are difficult to synthesise. In the case of 4EGI-1, activity requires 4E-BP1 expression.^48^ SBI-756 is a prototype of a novel class that binds to eIF4G and disrupts association of this scaffolding protein with eIF4E, independent of 4E-BP1. Active in cells in the mid-nanomolar range and in mouse models, this compound provides proof of concept for suppressing eIF4F function via blockade of key protein:protein interactions in the complex. Our data indicate that eIF4F-disrupting molecules like SBI-756 have great potential to sensitise lymphoma cells to venetoclax. Together with our previous finding that SBI-756 can circumvent resistance to BRAF inhibitors in melanoma, these results support further combination studies of SBI-756 with other targeted agents.

## Supplementary information

Supplementary Materials

## Data Availability

All data supporting the findings of this study are available within the article and its supplementary information files, or from the corresponding author on reasonable request. All RNA-seq data files along with their associated metadata have been deposited in the GEO database under the accession code GSE159906.
