# Supplementary material for: Targeting eIF4F translation initiation complex with SBI-756 sensitises B lymphoma cells to venetoclax
Source: Br J Cancer. 2020 Dec 14;124(6):1098–109. doi: 10.1038/s41416-020-01205-9 (PMC7960756; doi:10.1038/s41416-020-01205-9)
Supplement: Supplementary file 1 — Supplementary Materials [file 41416_2020_1205_MOESM1_ESM.pdf]

Supplemental Materials

Supplemental methods

Chemicals

We obtained rapamycin and MLN0128 from LC Laboratories (Woburn, MA, USA) and ABT-199 from Active Biochem (Wan Chai, Hong Kong). SBI-0640756 was synthesized as described (1) or purchased from Selleck with comparable results. Q-VD-OPh was obtained from SA (St. Louis, MO), and dimethyl sulfoxide (DMSO) from Fisher Scientifc (Waltham, MA, USA).

Cell culture

OCI-LY1, OCI-LY7, OCI-LY8, OCI-LY18, SU-DHL-4, SU-DHL-6, and VAL cell lines were obtained from Dr. Laura Pasqualucci (Columbia University, NY). These cell lines were validated by STR profiling (University of Arizona Genomics Core). Cells were cultured in IMDM (GE Healthcare Hyclone, Little Chalfont, UK) supplemented with 10% fetal bovine serum (FBS) (SA), 10 mM 4-(2-hydroxyethyl)-1-piperazineethanesulfonic acid (HEPES) (Corning), 10 mM L-Glutamine with 100 I.U. penicillin and 100 μg/ml streptomycin (Gemini Bio-products, Sacramento, CA). MAVER-1, JEKO-1, Mino, CCMCL1, and UPN-1 were kindly provided by Dr. Selina Chen-Kiang and Dr. Maurizio Di Liberto (Weill Cornell Medical College). These cells were cultured in RPMI-1640 (Corning) supplemented with 10% heat-inactivated FBS, 10 mM HEPES, 2 mM L-glutamine and 100 I.U./ml penicillin/streptomycin. Cells were grown in a humidified 37°C incubator with 5% CO2. Cells were routinely tested to ensure absence of mycoplasma and validated by STR profiling, and were maintained at or below 2x10e6 cells/ml. Human embryonic kidney (HEK) 293T cells were cultured in Dulbecco's Modified Eagle

Medium (DMEM; Life Technologies, Carlsbad, CA, USA) supplemented with 10% calf serum, 100 I.U. penicillin, and 100 μg/ml streptomycin. Human peripheral blood mononuclear cells (PBMCs) were isolated from blood samples by centrifugation through Ficoll-Paque™ (GE Healthcare, Piscataway, NJ, USA) and were grown in RPMI with 10% FBS, 10 mM HEPES, 10 mM L-Glutamine, 100 I.U. penicillin, 100 μg/ml streptomycin.

Immunoblotting

Cells were lysed in radio-immunoprecipitation assay buffer (150 mM NaCl, 1.0% IGEPAL® CA- 630, 0.5% sodium deoxycholate, 0.1% SDS, and 50 mM Tris, pH 8.0, 2 mM EDTA, 50 mM NaF) supplemented with protease inhibitor cocktail (Calbiochem, USA) and phosphatase inhibitor cocktails 2 and 3 (SA). Protein concentrations were normalized using a Bradford protein assay (Bio-Rad, Hercules, CA). Lysates were prepared at 1 μg/μl concentration in 1X XT Sample Buffer (Bio-Rad) and 5% BME (SA). Lysates were run on 8-12% Bis-Tris gels, and transferred onto nitrocellulose membranes (Bio-Rad). Antibodies to the following phosphoproteins and total proteins were used: phospho-Akt (S473), Akt, phospho-rS6 (S240/244), phospho-BAD (S136), phospho-4E-BP1 (Thr 37/46), 4E-BP1, 4E-BP2, GAPDH, Actin, PARP, caspase 9, cleaved caspase 3, MCL-1, BCL-2, BCL2L1(BCL-xL), survivin, eIF4E, eIF4G1, BIM, ERK1/2, and BAD (Cell Signaling Technology, Beverly, MA, USA), and NOXA (Abcam, Cambridge, MA). We used the anti-mouse IgG and anti-rabbit IgG secondary HRP-conjugated antibodies from Promega (Madison, WI, USA). Antibody dilutions were performed according to the manufacturer's instructions. Immunoreactive bands were visualized using Amersham ECL Prime Western Blotting Detection Reagent (GE Healthcare Life Sciences, Marlborough, MA) or Super Signal West Femto Maximum Sensitivity Substrate (Thermo Fisher

Scientific, Carlsbad, CA) and detected using a Nikon D700 SLR camera as described previously(2). Images were processed and densitometry was quantified using ImageJ software (NIH).

Cell viability

Cell viability assays were performed in 96-well U-bottom plates, with 6x10e4 cells in 200 µl. Cells were harvested by centrifuging the 96-well plate in a plate spinner centrifuge at 1700 rpm for 5 minutes. Cells were washed and stained with Annexin V conjugated to Alexa Fluor™ 647 nm (Thermo Fisher Scientific) 0.1mg/ml propidium iodide (PI)-staining solution (Life Technologies). Cells were then analyzed on a FACSCalibur flow cytometer (Becton-Dickinson, San Jose, CA) or ACEA NovoCyte Flow Cytometer (ACEA Biosciences, San Diego, CA) and cell cycle analysis performed using FlowJo software v.5.7.2 (TreeStar, Ashland, OR). Percentage of viable cells was determined based on the fraction of total cells that were Annexin V-negative and PI-negative.

Synergy calculation

Synergy between SBI-756 and venetoclax among GCB-DLBCL and MCL cells was perfomed as described previously(3). Briefly, predicted combination indexes for cells of interest treated with venetoclax and SBI-756 using combination index (CI) theorem of Chou-Talalay and CalcuSyn software (CI > 1 antagonism, CI = 1 additivity, CI < 1 synergy). Data were normalized to untreated control and converted to fractional effect as the inverse of viability (in other words: fractional effect of 0 is synonymous with 100% viability). Black circles are experimental data. Isobolograms produced for each cell line are presented.

Cell Cycle Analysis

6x10e6 cells were harvested, fixed in 50% ethanol, washed and stained in 0.1 mg/ml propidium iodide (PI)-staining solution (Life Technologies). Cells were then analyzed on a FACSCalibur flow cytometer (Becton-Dickinson), and cell cycle analysis performed using FlowJo software v.5.7.2 (TreeStar). After exclusion of cell debris, the induction of cell death was measured by calculating the percentage of intact cells with Sub-G1 DNA content.

Luciferase Reporter Assays to Measure Cap-Dependent Translation

Luciferase reporter construct pRSTF-CVB3 (kindly donated by Dr. Semler (UC Irvine)) containing the 5' NCR of the Coxsackie B3 virus(4) was cloned between a firefly (*Photinus pyralis)* and *Renilla* (*Renilla reniformis*) luciferase. The construct was used to measure cap-dependent translation as well as IRES dependent translation. For all experiments, cells were transfected in a FBS free media using Gene Pulser Xcell modular electroporation system (Bio-Rad) at 280V and 0.975 F. Cells were allowed to recover in complete IMDM media for three hours followed by inhibitor treatment for 16 hrs. Following treatment, cells were lysed and *Renilla* was measured, followed by firefly luciferase expression. Measurements were done using the Dual-luciferase assay kit (Promega) and by using a luminometer (Titertek-Berthold, Pforzheim, Germany). Results were expressed relative to untreated control.

Duolink Proximity Ligation Assay (PLA)

We performed PLA as described before (5). Briefly: 2x10e6 cells were treated for four hours as indicated. Cells were washed with 1x phosphate buffered saline (PBS) (Corning, NY) and

fixed with 4% paraformaldehyde (Thermo Fisher Scientific). CometSlides (Trevigen, Gaithersburg, MD) were coated with Poly-L-Lysine 0.1% solution (Sigma Aldrich (SA), St. Louis, MO), and cells were allowed to adhere. We followed the protocol of Duolink PLA (6); briefly: Cells were blocked using Duolink blocking solution, followed by probing with primary antibodies for eIF4G1 (Cell signaling Technologies, Danvers, MA, Cat. #2858, 1:200 dilution) and eIF4E (BD Biosciences, San Diego, CA, Cat. #610269, 2.5 μg/ml final). Next, cells were incubated with Duolink In Situ PLA Probe Anti-Rabbit PLUS (Cat. # DUO92002) and Duolink In Situ PLA Probe Anti-Mouse (Cat. # DUO92004) and allowed to ligate using ligation mix. Next, amplification and washes were performed as instructed and the slides were mounted using media containing DAPI. Slides were images using Leica TCS SP8 confocal microscope. Signal obtained was quantified using ImageJ software, and normalized to the number of cells per field (using DAPI nuclei staining). Images shown indicate the signal (Orange Duolink™) and nuclei for each field imaged, while graphs presented indicate ratio values of signal per cell in each field imaged.

Puromycin Incorporation Assay

5x10e6 cells were treated for 16 hours as indicated. In the last 15 minutes before harvesting, we added cycloheximide, puromycin (10 μg/ml) or vehicle to the controls. Additionally, we added puromycin (10 μg/ml) to all of the treatments to prevent protein elongation. Following a wash with PBS (Corning, NY), we fixed the cells with eBioscience™ Foxp3 / Transcription Factor Staining Buffer (Thermo Fisher Scientific, Carlsbad, CA) for 15 minutes. Following two washes with PBS-T (0.05-0.1% Tween-20), we stained the cells with diluted (1:100) anti-puromycin conjugated 488nm antibody (clone 12D10, Sigma Aldrich (SA), St. Louis, MO) and incubated

for 1 hour. Next, the cells were washes three times with PBS and analyzed on ACEA NovoCyte Flow Cytometer (ACEA Biosciences, San Diego, CA). Puromycin incorporation analysis performed using FlowJo software v.5.7.2 (TreeStar, Ashland, OR) looking for viable single cells and measuring the geometric mean of puromycin 488nm signal.

Quantitative Real Time PCR

RNA was extracted from cells using Trizol (Life Technologies), and 1 mg of RNA was reverse transcribed using the iScript cDNA synthesis kit (Bio-Rad). Gene specific primers were used to amplify MCL-1, Cyclin D3 and Actin using the Step One Real time PCR system and relative quantification of the transcripts was done using the delta delta cT (ΔΔCt) method. 4EBP1, 4EBP2 and actin specific primers were used in a 30 cycle PCR reaction using the S1000 thermal cycler (Biorad) to check for expression of the relevant mRNA transcripts.

RNA-seq and analysis

Fractions containing 4 or more ribosomes (considered well-translated) were pooled and RNA quality was measured by a Bioanalyzer (Agilent Technologies). RNA-seq was carried out by the New York University School of Medicine Genome Technology Core using the Illumina HiSeq 4000 single read. To examine differences in transcription and translation, total mRNA and polysome mRNA were quantile-normalized separately. Statistical analysis was performed using RIVET (Ernlund et al 2018. *BMC Genomics*). GO analysis was performed using the DAVID online tool.

Retro/lentiviral transductions

For all viral productions, HEK 293T cells were transfected using X-tremeGene HP DNA Transfection Reagent (Roche, Switzerland). 293T cells were incubated for 24 hours prior to replacing medium with IMDM. These virus-containing media were then harvested after an additional 24 hours and used to transduce DLBCL cell lines. For retroviral production, 293T cells were co-transfected with pCL-ampho viral packaging vector (Novus Biologicals, Littleton, CO, USA) whereas pCMV-VSV-G (Addgene plasmid 8454) and psPAX2 (Addgene plasmid 12260) were co-transfected for lentivirus production. To transduce DLBCL cell lines, we incubated cells in viral supernatants for 72 hours (changing supernatant every 24 hours) with 10 μg/ml 1,5-dimethyl-1,5-diazaundecamethylene polymethobromide (polybrene, SA). Cells were treated with either blasticidin (8 μg/ml) or puromycin (2 μg/ml) for 5 days after transduction to select for stably transduced cells. Plasmid positive cells were maintained with blasticidin (4 μg/ml) or puromycin (1 μg/ml).

Generation of Cell Lines with Inducible Expression of Wild Type 4E-BP1 or 5A Mutated 4E-BP1

To generate DLBCL cells with doxycycline-inducible expression of a gene of interest, cells were first transduced with pMA2640 (Addgene plasmid #25434) and selected for blasticidin resistance. Expression of the improved tetracycline-controlled transactivator (rtTA-Advanced) allowed for doxycycline-inducible expression of genes downstream of the modified Tet-responsive element provided in the pLVX-tight-puro vector (Takara). Full length rat wild type 4E-BP1 was obtained from Dr. John Lawrence (University of Virginia, Charlottesville, VA). The 4E-BP1 construct in which five phosphorylation sites (all Serine/Threonine phosphorylation

sites) were changed to Alanine ("5A mutant") was a kind gift from Dr. Davide Ruggero. These constructs were cloned into the pLVX-Tight-Puro Dox inducible system using NotI and EcoRI restriction sites. High titer lentivirus was produced using the lentiviral packing and envelope constructs mentioned above. OCI-LY1 and OCI-LY8 cell lines expressing the reverse tetracycline transactivator protein (rtTA) were infected with the lentivirus and selected in IMDM culture medium containing 8 μg/ml Blasticidin and 2.0 μg/ml Puromycin. The wildtype (or mutant) 4E-BP1 expression was induced by addition of 1 μg/ml Doxycycline for 16–24 hrs. All pLVX-tight-puro plasmids were sequenced using the following primer, 5'-AGCTCGTTTAGTGAACCGTCAGATC-3'.

Generation of OCI-LY1 4EBP1 and 4EBP2 KO Cell Lines

To generate OCI-LY1 cells genetically edited to express CRISPR/Cas9 system that knocks out (KO) 4EBP1, the lentiviral vector lentiCRISPRv2-puro (Addgene plasmid #98290) was processed to contain 4EBP1 guided sgRNA. We used two different sgRNA sequences: CACCGGAGCACCACCCGGCGAGTGG (sgRNA1) and CACCGGGGCTCATCACTGGAAGGGC (sgRNA2). Transduced cells were selected for puromycin resistance and cloned cell lines validated for 4EBP1 KO via western blot analysis (Fig. S6).

Generation of OCI-LY1 cell line with SBI-756 resistance

OCI-LY1 cells were cultured as described above. We introduced the cells to increasing SBI-756 concentrations in their culture media with every cell passaging (from 10 nM to 1.1 μM).

Statistical analysis

The number "n" of biological replicates for each experiment is indicated in the figure legends. Two-way ANOVA for multiple comparisons was performed where indicated while considering sample independence, variance equality and normality. Student *t*-tests were applied to population means assuming equal variance (standard deviations within two-fold). The use of one- versus two-sample tests, and paired versus unpaired comparisons, was justified by the experimental design as indicated in the Figure Legends.

Supplemental Bibliography

1. Feng Y, Pinkerton AB, Hulea L, Zhang T, Davies MA, Grotegut S, et al. SBI-0640756 Attenuates the Growth of Clinically Unresponsive Melanomas by Disrupting the eIF4F Translation Initiation Complex. Cancer Res. 2015 Dec 15;75(24):5211–5218.
2. Khoury MK, Parker I, Aswad DW. Acquisition of chemiluminescent signals from immunoblots with a digital single-lens reflex camera. Anal Biochem. 2010 Feb 1;397(1):129–131.
3. Chou T-C. Drug combination studies and their synergy quantification using the Chou-Talalay method. Cancer Res. 2010 Jan 15;70(2):440–446.
4. Jang GM, Leong LE-C, Hoang LT, Wang PH, Gutman GA, Semler BL. Structurally distinct elements mediate internal ribosome entry within the 5'-noncoding region of a voltage-gated potassium channel mRNA. J Biol Chem. 2004 Nov 12;279(46):47419–47430.
5. Chiu H, Jackson LV, Oh KI, Mai A, Ronai ZA, Ruggero D, et al. The mTORC1/4E-BP/eIF4E Axis Promotes Antibody Class Switching in B Lymphocytes. J Immunol. 2019 Jan 15;202(2):579–590.
6. Boussemart L, Malka-Mahieu H, Girault I, Allard D, Hemmingsson O, Tomasic G, et al. eIF4F is a nexus of resistance to anti-BRAF and anti-MEK cancer therapies. Nature. 2014 Sep 4;513(7516):105–109.

Supplemental Figure Legends

Supplemental Figure 1: Constitutively active 4E-BP1 mutant sensitizes lymphoma cells to venetoclax and navitoclax, similar to the effect of TOR-KI

(A) Western blot analysis of OCI-LY1 cells expressing rtTA and treated with doxycycline. EV = cells expressing normal levels of 4E-BP1; WT = cell expressing normal 4E-BP1 form. Mut = cells expressing constitutively active 4E-BP1-5A mutant. Cells were doxycycline treated with or without MLN0128 (TOR-KI) or BEZ235 (dual PI3K/mTOR inhibitor) for 16 hours. We performed western blot analysis for mTOR main substrates, with total AKT serving as a loading control.

(B) OCI-LY1 cells EV, WT, Mut cells (as described in A) were treated with doxycycline with or without MLN0128 or BEZ235, in combination with navitoclax (ABT-263). We assessed cells' viability by measuring Annexin V and propidium iodide (PI) staining, and compared the IC_{50} of navitoclax under different conditions. Data are plotted for individual experiments with the means of each group indicated by a horizontal line. *$p<0.05$; **$p<0.01$; ****$p<0.001$, vs vector, unpaired *t*-tests.

Supplemental Figure 2: SBI-756 treatment prevents eIF4E:eIF4G1 interaction in a dose-dependent manner

(A, B) OCI-LY8 DLBCL cells were tested for eIF4E:eIF4G1 association via PLA and analyzed as described in Figure 1A, B. (**A**) Scale bar = 33µm. Representative images of at least three fields are shown. **(B)** Data are plotted for individual experiments with the means of each group indicated by a horizontal line. * $p<0.05$; ** $p< 0.01$. Paired one-sample *t*-test. **(C-E)** Cap-

dependent translation was measured in **(C)** GCB-DLBCL (OCI-LY8) or **(D)** MCL (MAVER-1) or **(E)** GCB-DLBCL (SU-DHL-6) cells as described in Figure 2C. *p<0.05, **p<0.01, ***p<0.005. One-sample *t*-test vs. DMSO control. n = 3.

Supplemental Figure 3: SBI-756 does not change mTOR substrate phosphorylation

OCI-LY1, OCI-LY8 or VAL DLBCL cells were treated with vehicle (DMSO), MLN0128, rapamycin or increasing SBI-756 concentration. Phosphorylation of canonical mTORC1 and mTORC2 substrates was analyzed by western blot analysis **(A, C)** and quantified relative to vehicle treated cells **(B, D)**. The results were quantified using ImageJ by a blinded observer. ***p<0.005, ****p<0.001. One-sample *t*-test vs. normalized control, n = 3.

Supplemental Figure 4: Sensitization of DLBCL and MCL cells to venetoclax treatment by targeting eIF4F complex

GCB DLBCL cell lines **(A)** or MCL cell lines **(B)** were treated for 48 hours with increasing v*enetocla*x concentrations in combination with vehicle (DMSO) control or various inhibitors as indicated. Cell lines tested are indicated in the title of each graph. Viability was assessed using Annexin V and PI staining. We performed unpaired *t*-tests and compared each treatment group to vehicle treated group. *p<0.05; **p<0.01; ****p<0.001, adjusted for multiple comparisons. **(C)** We calculated IC_{50} values for each cell line tested based on the viability assays performed. Isobologram plots were graphed based on Chou-Talalay method for synergy calculation (combination index)[11] using median effect method for cell lines treated for 48 hours with combinations of SBI-756 and venetoclax at fixed ratios. GCB-DLBCL: OCI-LY8, SU-DHL-6, OCI-LY18, and MCL: MAVER-1 cell lines were tested. **(D)** We treated OCI-LY1 parental, OCI-

LY1 that were selected for SBI-756 resistance or OCI-LY7 cells for 4 hours with either DMSO (vehicle) control or SBI-756 250 nM. The cells were fixed and eIF4E:eIF4G1 association was analyzed using PLA, as described in figure 1A, B. We quantified the results using ImageJ software and normalized to vehicle treatment for each of the cell lines. * $p<0.05$; ** $p< 0.01$. Paired one-sample *t*-test.

Supplemental Figure 5: SBI-756 in combination with venetoclax induces apoptosis

(A) OCI-LY1 and OCI-LY8 cells were treated with vehicle (DMSO), MLN0128, venetoclax or increasing SBI-756 concentration for 16 hours. We also tested combinational treatments of venetoclax with MLN0128 or together with SBI-756. Cleavage of PARP or caspase 3 were evaluated as indications of apoptosis induction via western blot analysis. Beta actin served as a loading control.

(B) OCI-LY1 or OCI-LY8 cells were treated for 48 hours with vehicle (DMSO) control or various inhibitors as indicated. Viability of the cells was tested in the presence or absence of a pan caspase inhibitor (Q-VD-OPh hydrate, 20μM). Viability was assessed using Annexin V and PI staining. *$p<0.05$; **$p<0.01$; ****$p<0.001$, unpaired *t*-tests comparing each treatment group to vehicle treated group, and adjusted for multiple comparisons.

Supplemental Figure 6: 4E-BP1 KO clones are insensitive to TOR-KI treatment, yet remain sensitive to SBI-756 treatment

(A) We performed a western blot analysis to validate OCI-LY1 CRISPR/Cas9 clones produced. The cells were lysed and ran on an SDS-PAGE gel, followed by probing for the targets indicted. CRISPR/Cas9 clones were produced by transfection of OCI-LY1 cells stably expressing Cas9

system with guide RNAs. Clones were isolated that carried an empty vector; or sgRNAs specific for 4E-BP1 (*4E-BP1 KO*). For subsequent experiments we used clones with validated loss of 4E-BP1 expression (sgRNA 1-1, 1-2, 2-1). **(B-C)** OCI-LY1 cells stably expressing Cas9 were transfected with guide RNAs. Clones were isolated that carried an empty vector (EV); or sgRNAs specific for 4E-BP1 (*4E-BP1 KO*). Cells were treated for 48 hours with titrated amounts of venetoclax without (vehicle) or with MLN0128 **(B)** or SBI-756 **(C)**. Viability was assessed using annexin V and PI staining. *p<0.05, ns = not significant. Two way ANOVA vs. control (EV).

Supplemental Figure 7: Additional data from *in vivo* testing of venetoclax and SBI-756

(A) Body weight of each mouse was measured daily. Average body weight for each treatment group is presented as mean +/- SEM. *p<0.05, **p<0.01, ***P<0.005, ****p<0.001, ANOVA, P value has been adjusted for multiple comparisons (using Tukey's adjustment for multiple comparisons).

(B) The weight of each tumor was measured at the day of sacrifice (following five days of treatment) and plotted as individual measurements with the mean (horizontal line). *p<0.05, **p<0.01, ***P<0.005, ****p<0.001, ANOVA, P value has been adjusted for multiple comparisons (using Tukey's adjustment for multiple comparisons)

(C) Tumor volume was measured at the day of sacrifice and plotted as individual measurements with the mean (horizontal line). *p<0.05, **p<0.01, ***P<0.005, ****p<0.001, ANOVA, P value has been adjusted for multiple comparisons (using Tukey's adjustment for multiple comparisons)

(D) and **(E)** Tumors were excised from euthanized mice and dissociated into single cells. Cells were fixed and immune-stained for canonical mTOR substrates: phosphorylated S6 and phosphorylated 4E-BP1. Phosphorylation levels were evaluated using flow cytometry and quantification of geometric mean (G-mean). Controls (D) consisted of cell lines treated with the inhibitors indicated and tested for phosphorylation of targets mentioned. In vivo samples (E) consisted of cells excised from tumors from each treatment group, and immune-stained for the same targets.

Supplemental Figure 8: SBI-756 induces a decrease in MCL-1, BCL-xL, survivin, eIF4E and eIF4G

(A, B) OCI-LY1 cells were starved for 24 hours in media containing 1% FBS, then restored to complete media (10% FBS) in the presence of vehicle (DMSO) or the indicated concentrations of MLN0128 or SBI756 for 4 hours. Lysates were prepared and subjected to western blot analysis with antibodies to the proteins shown on the left. Band intensities were determined by ImageJ and normalized to the loading control (GAPDH). Panel **A** shows data from a representative Western blot experiment. Panel **B** shows quantitation over multiple experiments. *$p<0.05$, **$p<0.01$, ****$p<0.001$. One-sample *t*-test vs. normalized control.

(C) OCI-LY1 cells were serum starved for 24 hours, followed by 4 hours of treatment with the inhibitors indicated. Next, we isolated RNA using Trizol-LS, and we synthesized cDNA by reverse transcription. Using unique primer sets for each target, we examined transcription levels of candidates among the samples isolated via real-time PCR. Based on the C_T values obtained, we calculated $\Delta\Delta C_T$ values that reflected the expression levels of the targets. Two-way ANOVA vs. control.

Supplemental Figure 9:

(**A**) Peripheral blood mononuclear cells (PBMCs) were obtained from healthy blood donors and were isolated as described previously[116]. PBMCs (mean +/- SD, n=3) were cultured for 48 hours with MLN0128 (30 nM) or SBI-756 (250 nM). One-sample *t*-test vs. DMSO control. n = 3. *p<0.05, **p<0.01, ***p<0.005 vs. DMSO control. Leukocyte subsets were distinguished by surface markers using flow cytometry). **(B)** OCI-LY1 cells were treated for 48 hours with increasing S63845 (MCL-1 inhibitor) concentrations in combination with vehicle (DMSO) control or various inhibitors as indicated. Cell lines tested are indicated in the title of each graph. Viability was assessed using Annexin V and PI staining. We performed unpaired *t*-tests and compared each treatment group to vehicle treated group. *p<0.05; **p<0.01; ****p<0.001, adjusted for multiple comparisons.

Supplemental Figure 10: Model for mechanism of venetoclax sensitization by SBI-756

SBI-756 prevents eIF4E-eIF4G1 interaction and formation of translation initiation complex. venetoclax induces apoptosis by targeting BCL-2, a critical anti-apoptotic protein.

(**A**) Diagram of mTOR signaling pathway and targets of the inhibitors used in this study.

(**B**) Diagram of gene ontology groups whose translation efficiency is reduced 4hr after treatment of lymphoma cells with SBI-756. Collectively, these selective changes in translation of mRNAs lead over time to reduced protein synthesis rate.

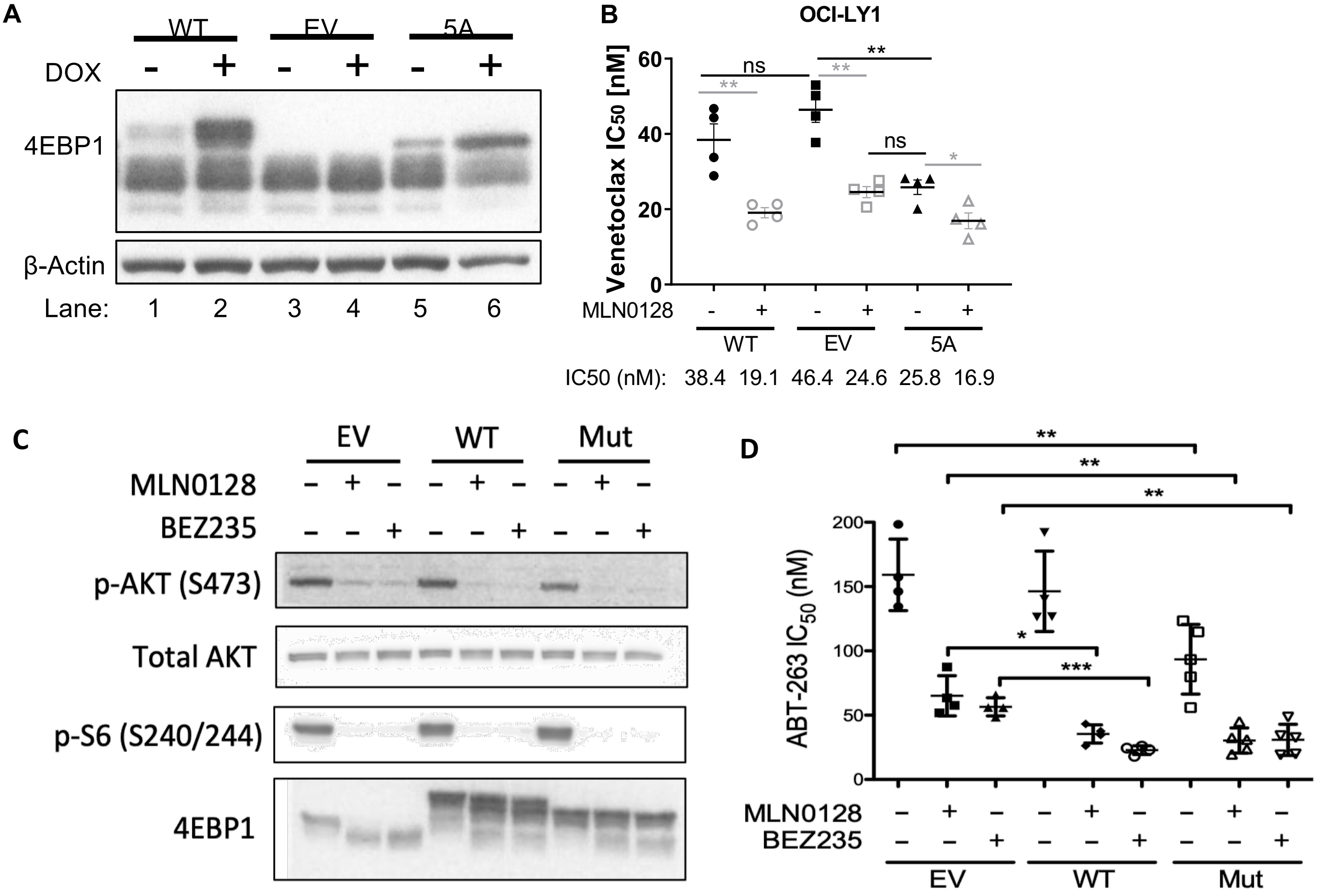

Supplementary Figure 1

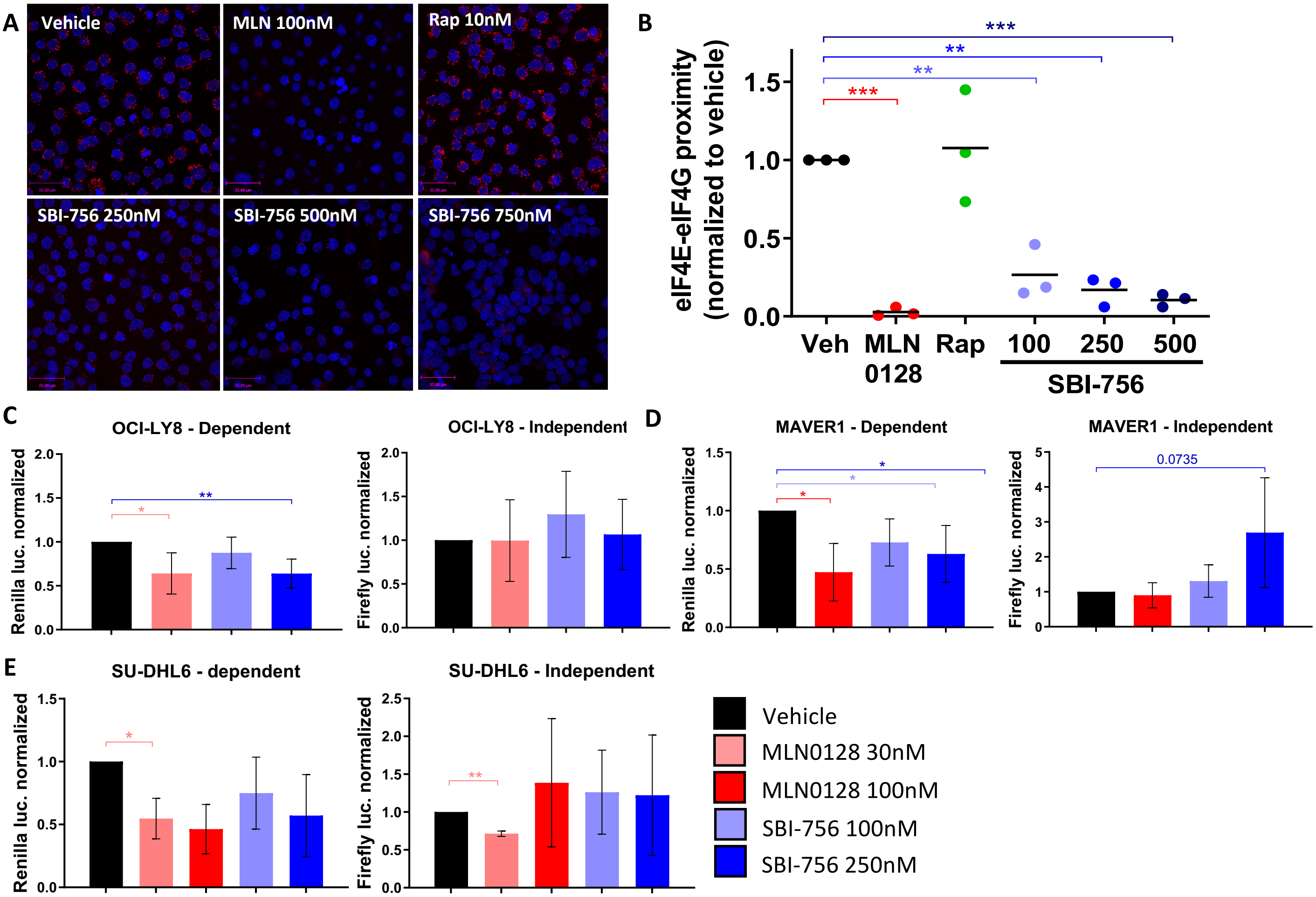

Supplementary Figure 2

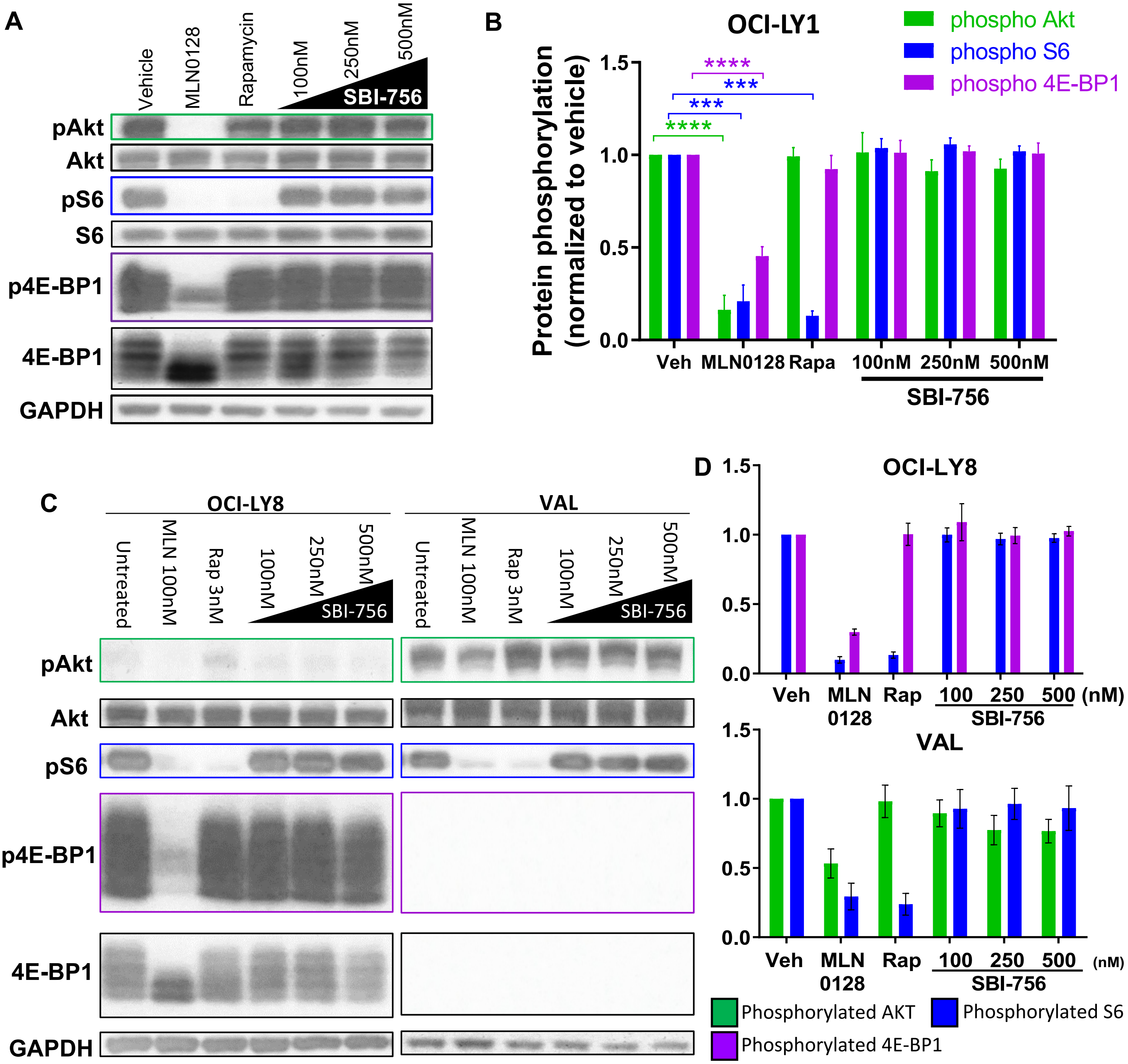

Supplementary Figure 3

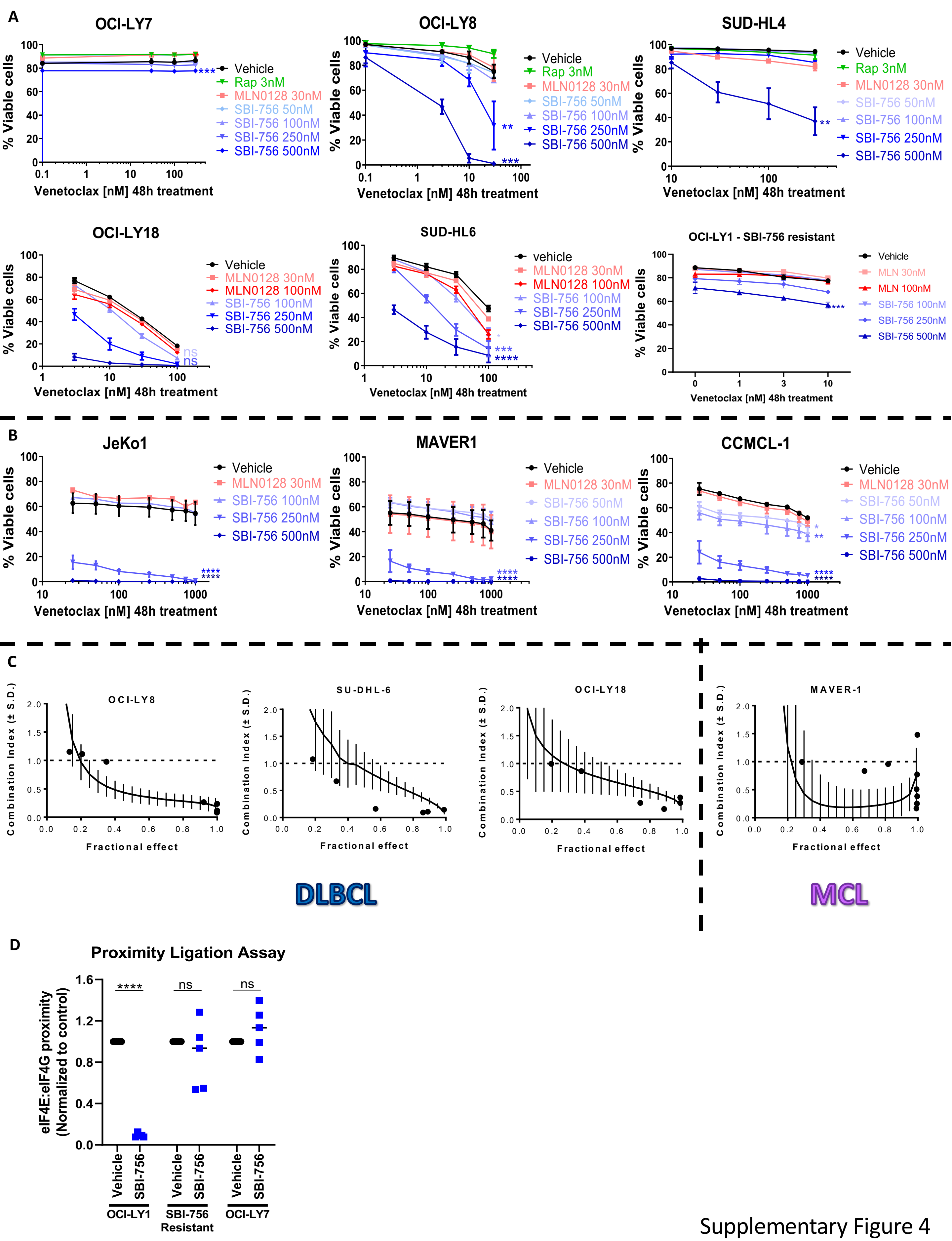

Supplementary Figure 4

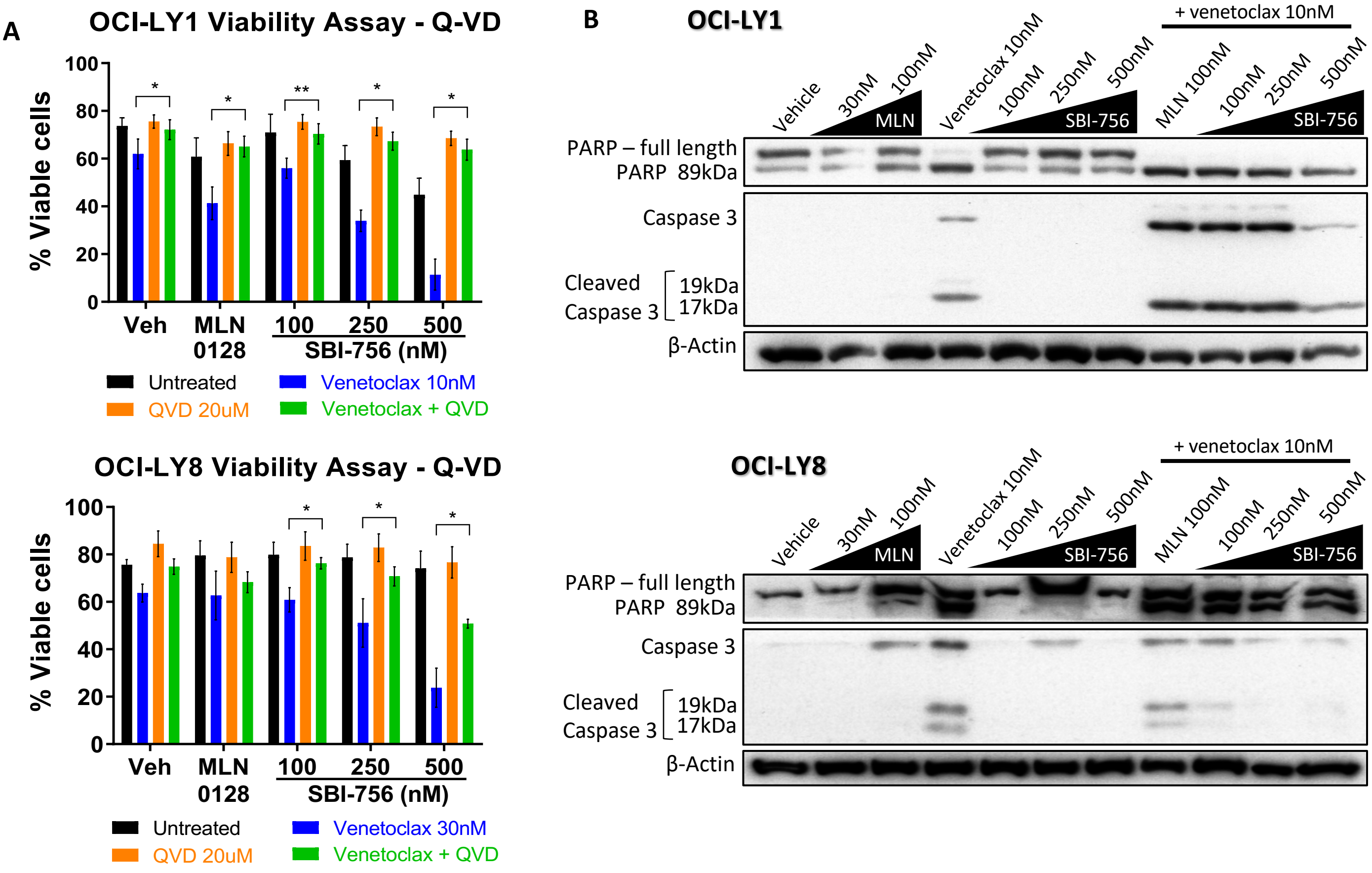

Supplementary Figure 5

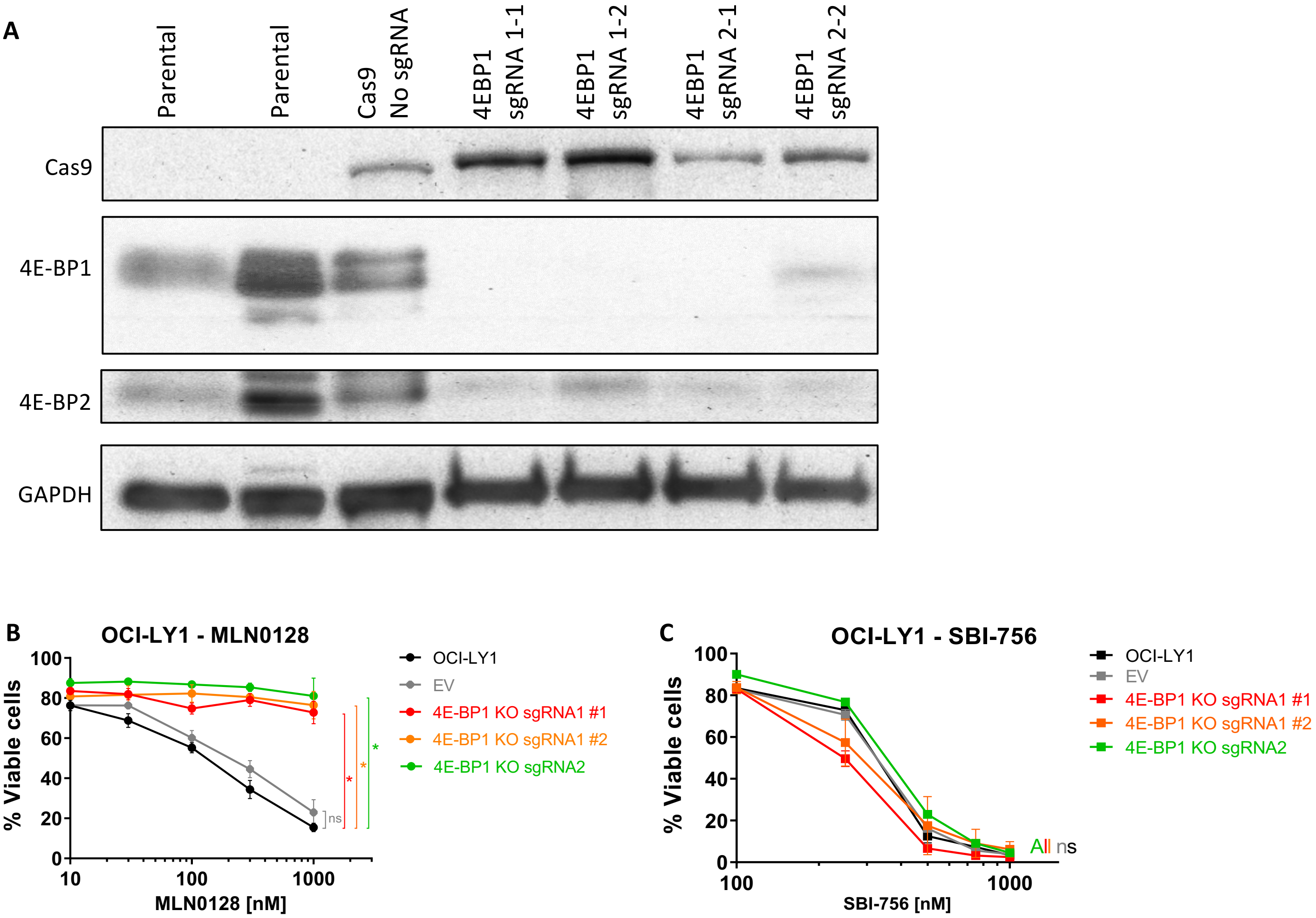

Supplementary Figure 6

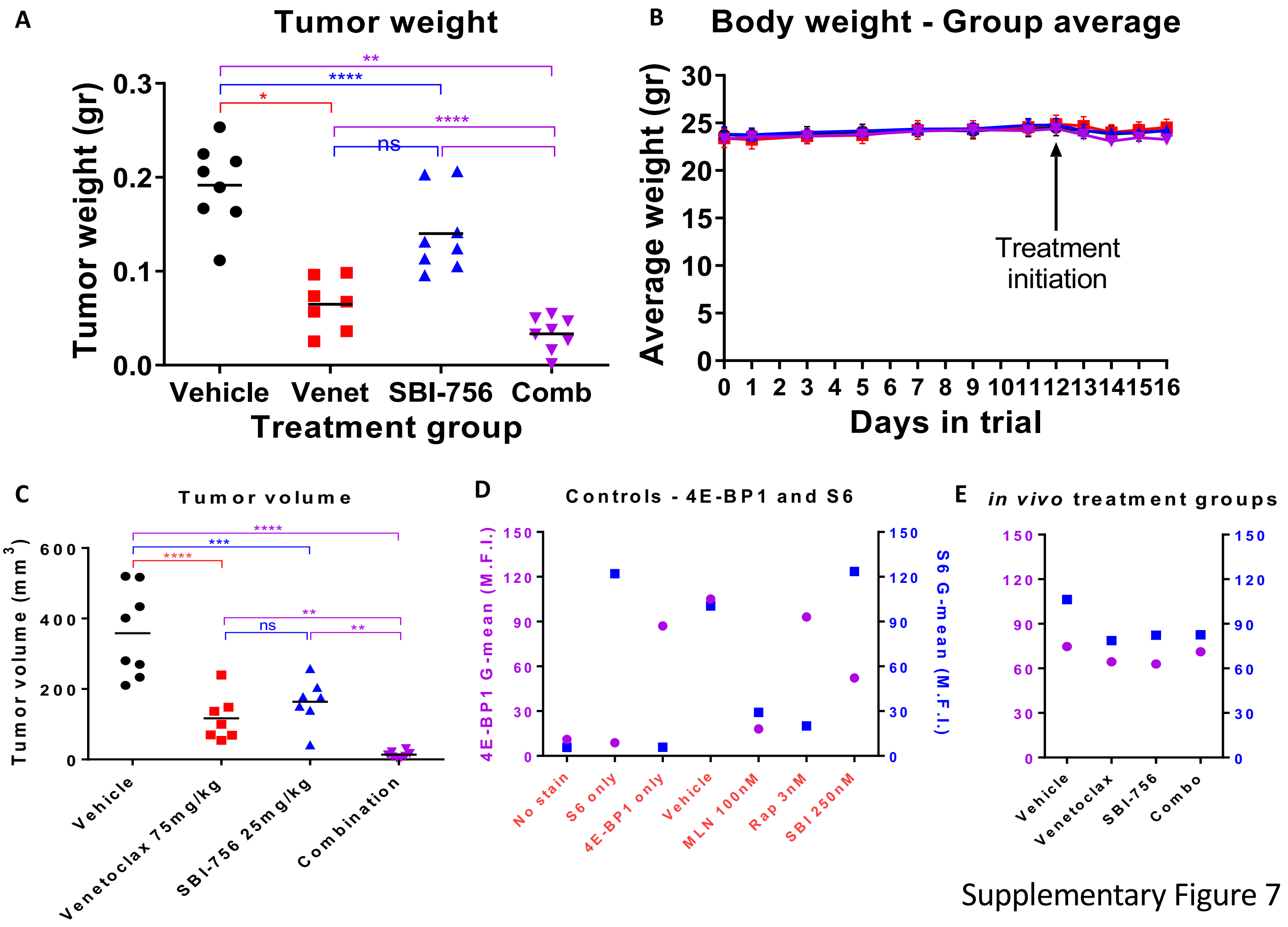

Supplementary Figure 7

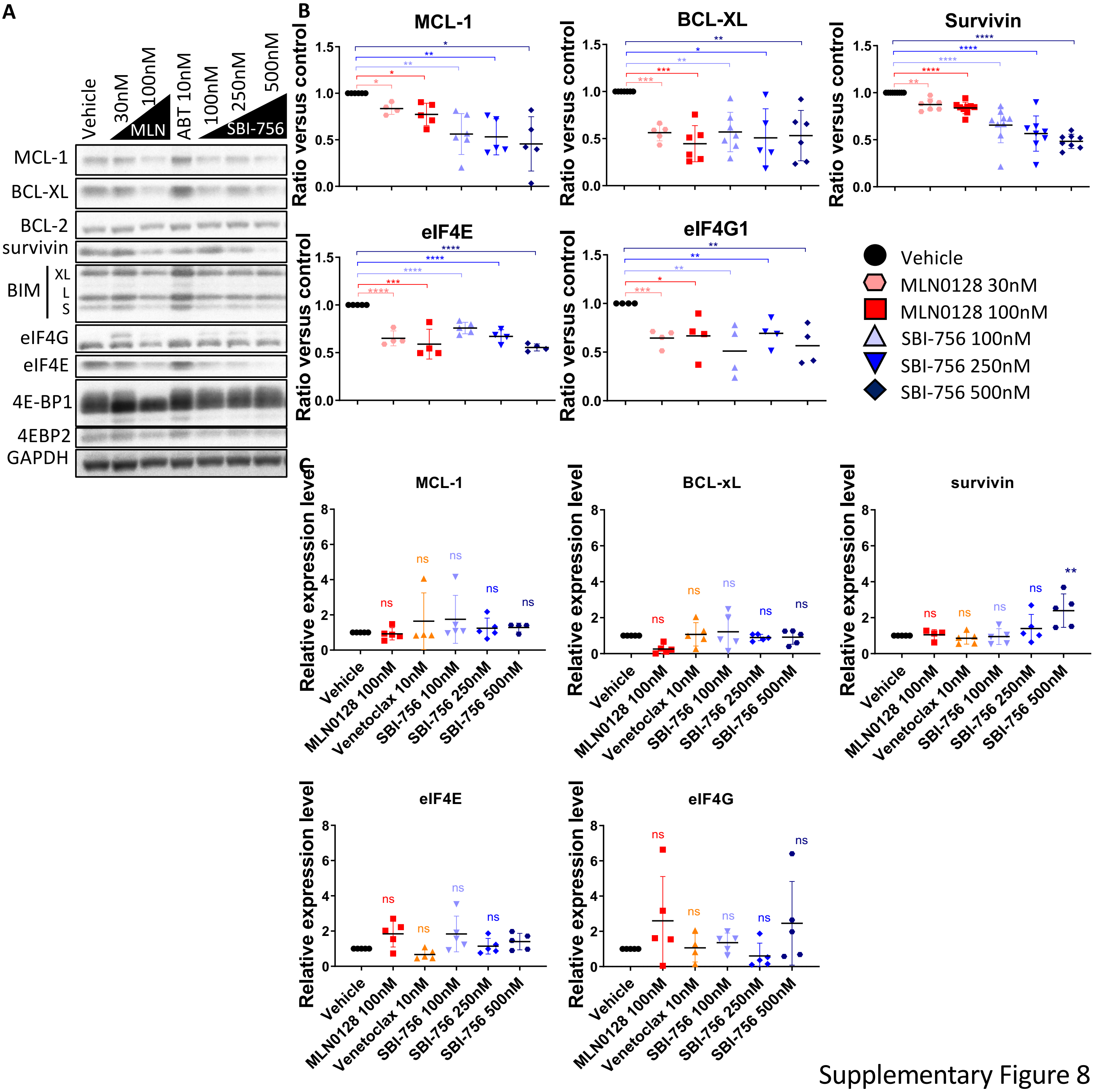

Supplementary Figure 8

A

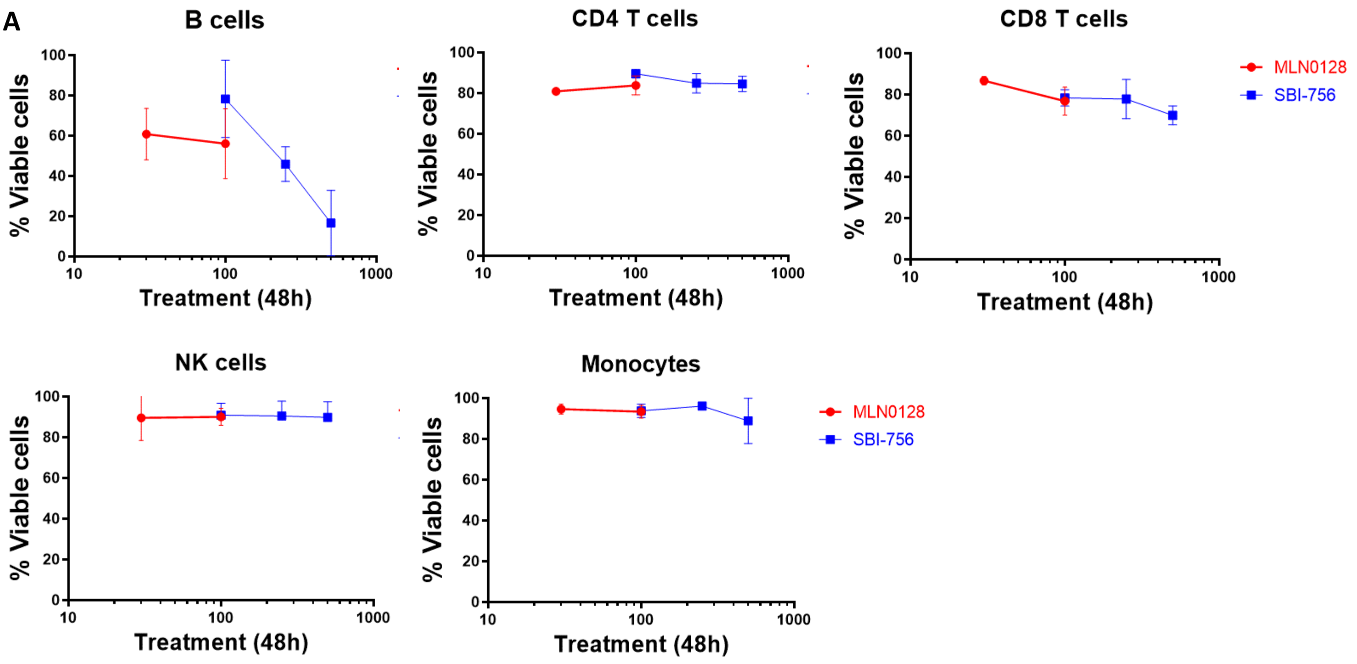
B cells
CD4 T cells
CD8 T cells
NK cells
Monocytes
% Viable cells
Treatment (48h)
MLN0128
SBI-756
10
100
1000
0
20
40
60
80
100

B

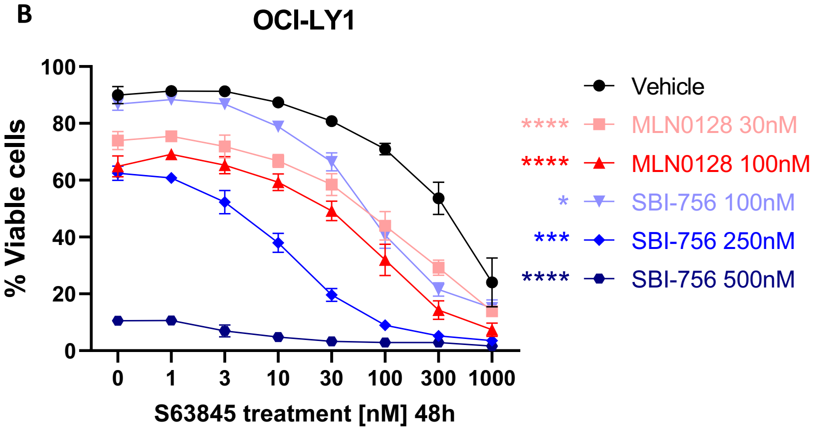
OCI-LY1
% Viable cells
S63845 treatment [nM] 48h
0
1
3
10
30
100
300
1000
Vehicle
**** MLN0128 30nM
**** MLN0128 100nM
* SBI-756 100nM
*** SBI-756 250nM
**** SBI-756 500nM

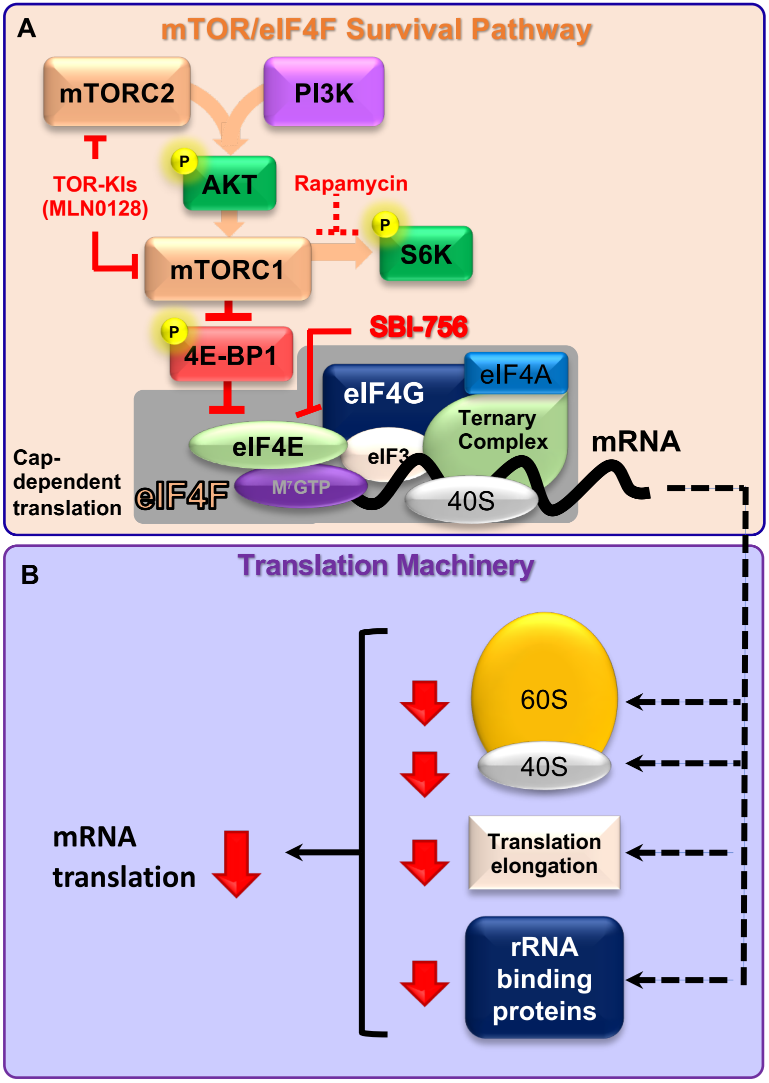

Supplemental Figure 10

Supplemental Table 1

Translationally down-regulated genes

Gene Name	Log Fold Change	p-value
MIR1282	-2.295724225	8.45E-05
PRRT1	-1.561692113	0.000252575
AC007387.2	-1.978255428	0.000708242
RP11-499E18.1	-1.709520223	0.000745384
RP5-1039K5.19	-1.29711273	0.000794354
CRIP1P2	-1.490962784	0.000848024
RP11-54O7.10	-1.852933428	0.00086131
SNHG9	-1.711127012	0.000885696
RPL18AP16	-1.369876008	0.001005552
AC253572.1	-1.485216348	0.001179318
MADCAM1	-1.183637342	0.001181126
MSX1	-1.847977164	0.001226067
RP11-346C16.4	-1.413735302	0.001390072
RP11-345K20.2	-1.351334391	0.001543558
RP11-304L19.2	-1.33540326	0.001563951
RPL29P7	-1.64144483	0.001593017
OR7E55P	-1.31294894	0.001597694
RP4-545L17.11	-1.94441718	0.001605258
AC016700.6	-1.278153654	0.001728788
HIST1H2BG	-1.125981016	0.002020867
RP1-101A2.1	-1.168330458	0.002154575
RPLP0P2	-1.48870102	0.002453323
RP11-524O24.2	-1.207248454	0.00245487
CTA-276O3.4	-1.5673522	0.002528948
RPL39P5	-1.306691669	0.002634601
TMSB4XP2	-1.296638584	0.002652612
OSER1-AS1	-1.005905418	0.002710646
HIST1H2BD	-1.646868477	0.002720944
AC012671.2	-1.433792734	0.002721091
RPL26	-1.221786781	0.002898714
RPL5P24	-1.067329141	0.002994559
RPS4XP7	-1.047476495	0.003019911
C16orf74	-1.777435719	0.003037182
RP11-50D9.1	-1.156422867	0.003106228
BRI3	-1.119175559	0.003167264
FTLP2	-1.182125255	0.003175842
RPL39P3	-1.397490655	0.003186353
RPL23AP38	-1.348219539	0.00321384
RP11-742D12.1	-1.507298027	0.003264851
RP11-192C21.1	-1.265654676	0.003359796
RNU1-1	-1.984964559	0.00336294

HIST1H4J	-1.450564292	0.003406681
AC010733.5	-1.083949677	0.003422932
RP11-434H6.7	-2.08554972	0.003476899
RP11-422P24.9	-1.072475566	0.003542096
RP11-307O1.1	-1.385559179	0.003556246
AC093673.5	-1.05137968	0.003576323
MYL6BP1	-1.062235407	0.003696272
TMSB4XP4	-1.597028633	0.003741049
DOC2GP	-1.283733322	0.003893544
CD248	-1.107783504	0.003953049
AC104978.1	-1.047230934	0.004066319
RP11-796G6.1	-1.131163583	0.004122659
FTL	-1.119266465	0.00418154
RBM34	-1.590932953	0.004239661
RPL30P4	-1.118476455	0.004266617
RPS28P7	-1.185514015	0.004270448
RP11-104D3.2	-1.181852732	0.004273991
RP4-631H13.6	-1.102155914	0.004302778
GH1	-1.568171013	0.004356383
MT-RNR1	-1.309345583	0.004373787
EIF1P5	-1.004015222	0.004392811
RP11-452G18.2	-1.319577117	0.004505786
RPS15AP11	-1.001300793	0.004522319
AC092933.3	-1.866956892	0.004681421
CTD-3184A7.4	-1.387076496	0.004750693
RP4-798A10.4	-1.189098982	0.004769805
CTD-2090I13.1	-1.660158222	0.005024859
RP11-40C6.2	-1.236169353	0.00505129
AC017104.6	-1.528218289	0.005140015
RP11-93H24.3	-1.606169555	0.005169616
MT2A	-1.189108283	0.005209378
RPS4XP20	-1.068031663	0.005216205
AC006128.2	-1.592947079	0.005231689
RP11-3P17.5	-1.891698672	0.005249762
TMSB4XP1	-1.120044954	0.005304087
TMSB4XP6	-1.183242321	0.005345627
RP11-2C24.9	-1.104114651	0.005361209
RN7SL4P	-1.914666674	0.005362447
MTRNR2L1	-1.280334725	0.005450552
RPL41P2	-1.13309594	0.00545981
RP11-586D5.3	-1.783167227	0.00550817
RP5-837I24.5	-1.036280896	0.005509506
RP4-798A10.7	-1.242585018	0.00560144
RPL23AP43	-1.16217457	0.005617286
RP11-389O22.4	-1.07451446	0.005658363

C6orf226	-1.109356744	0.005659521
RP11-66N11.8	-1.785157308	0.005780349
RP11-14K2.1	-1.092741323	0.005863316
SH3D19	-1.48175042	0.005891669
MTRNR2L10	-1.394930856	0.005913681
AC007389.2	-1.052692189	0.005969282
RP11-543B16.1	-1.108751341	0.00597769
SNHG25	-1.843767985	0.005989136
RPS15AP12	-1.120229871	0.006037918
RP11-302F12.1	-1.345392258	0.006044813
RP11-627K11.1	-1.370054445	0.006071306
RPL13AP6	-1.535030266	0.006093276
RPL30P7	-1.252018057	0.006139549
RPS19P3	-1.1945495	0.006181452
RPS15AP38	-1.082417911	0.006248638
CLDND2	-1.966726767	0.006275254
RP11-867G23.10	-1.516170969	0.006399911
RP11-377K22.2	-1.316711864	0.006408759
RP11-274J15.2	-1.215032374	0.006490189
RP11-701P16.5	-1.486754389	0.006492331
MTRNR2L6	-1.223841749	0.006496546
RP11-447H19.4	-1.052579424	0.006523294
CTD-3214H19.12	-1.059414337	0.006533099
RPL37A	-1.030965438	0.006534681
RP11-140K17.3	-1.33087852	0.006559459
RP11-566K19.6	-1.018078132	0.006572672
RP11-318C24.1	-1.383915468	0.006606691
RP11-253E3.1	-1.177977903	0.006663123
RP5-882O7.1	-1.338352104	0.006707232
AC016712.2	-1.01981375	0.006726243
ZFAS1	-1.198475849	0.006787806
RP11-404K5.1	-1.078716213	0.006796741
RP11-572P18.1	-1.506702482	0.006838773
HIST1H1C	-1.805879227	0.006842274
RP11-553K8.2	-1.469590889	0.006855152
DDIT3	-1.098452518	0.006878552
RP11-389O22.5	-1.390139605	0.006887902
CYP2W1	-1.270925277	0.006967062
RPL17P20	-1.143119274	0.006984071
RPS15AP17	-1.170649761	0.007107781
AC004453.8	-1.05325374	0.007178945
RPL26P35	-1.083540413	0.007255514
RPS29	-1.410040781	0.007297621
WEE2-AS1	-1.09812025	0.007327751
RPL13AP20	-1.684503327	0.007418921

RPL37P2	-1.003194145	0.007456965
RP11-397E7.1	-1.020773813	0.007501586
AURKC	-1.61469539	0.007616915
RP3-340B19.2	-1.055088592	0.007636848
RPL38	-1.014212301	0.007644851
RP11-332E4.1	-1.717243566	0.007668116
RP11-235D19.2	-1.085338599	0.007671869
RPL36	-1.078638162	0.007705998
EMP3	-1.18590495	0.007708391
RP11-264F23.1	-1.112507073	0.007740455
TMSB4X	-1.128875694	0.007792098
RP11-247I13.3	-1.429937371	0.007797327
RP11-661A12.8	-1.493926196	0.007851535
RP4-717I23.2	-1.235345081	0.007884725
SYCE1L	-1.324497144	0.007890656
DNAJC27-AS1	-1.84758347	0.007911192
FTLP17	-1.125433412	0.008052705
HSP90B1	-1.26095622	0.008053257
RPL32	-1.091852366	0.008080505
RPL37P6	-1.044584638	0.008109107
RP11-403P14.1	-1.223358887	0.008170455
VEGFB	-1.002383106	0.008206057
AC105399.2	-1.097733828	0.008356754
RPS3P6	-1.226883553	0.00836201
AC010095.7	-1.227179849	0.008364867
RPL41	-1.177385405	0.00847614
RP11-378J18.6	-1.339248978	0.008483095
RP11-244J10.1	-1.288901154	0.008623137
RP11-331N16.1	-1.098070489	0.008648557
AC017080.1	-1.471785384	0.00869148
MTRNR2L8	-1.02013928	0.008815627
CTB-129O4.1	-1.437427817	0.008830838
CTD-2031P19.4	-1.231655807	0.008901275
RP11-58A11.2	-1.133635625	0.008990273
SNHG8	-1.030656868	0.00904727
VGF	-1.019337037	0.009203055
RPS21	-1.086615567	0.00927073
RPS18P6	-1.017386213	0.009308248
ARMC12	-1.096128254	0.009464978
RPS2P35	-1.193771051	0.00950343
TMSB10	-1.209988055	0.009734753
RP11-440L14.1	-1.059618	0.009837021
CRIP1	-1.135608542	0.0098944
GAS5	-1.09189156	0.009924247
RPS27	-1.034478129	0.010034229

RPL29	-1.147168103	0.010050243
RP3-337H4.6	-1.44038193	0.010054357
RPS4XP11	-1.129716107	0.010228053
RPL35AP21	-1.1188308	0.01034785
RPS11	-1.043697017	0.010424555
RP5-940J5.9	-1.940716586	0.010442833
AC005019.2	-1.062996946	0.010465353
FTH1P10	-1.16273643	0.010515668
HNRNPUP1	-1.019533494	0.010592565
RP11-445N18.3	-1.428651363	0.010712662
RPL5P10	-1.069514968	0.010756611
RPS17P2	-1.255907539	0.010876183
RP11-114H7.1	-1.108037826	0.010878178
HSP90B3P	-1.087498502	0.010883702
RPL23AP57	-1.119473588	0.010970545
RP11-345J4.4	-1.314659472	0.011075721
LGALS1	-1.485881236	0.011216031
RPL28	-1.02809508	0.011316421
RP11-313M3.1	-1.036677051	0.011371894
NGFR	-1.381112596	0.011645579
TMEM91	-1.357788586	0.011661851
RP11-289K10.1	-1.2409972	0.011738099
RPL18P13	-1.034907764	0.011764075
IFITM4P	-1.524729581	0.01185913
LMNA	-1.139544074	0.011887079
CRIP1P4	-1.216021704	0.011900756
HSP90B2P	-1.225555547	0.011967577
AC079250.1	-1.182704984	0.011971778
RPL6P25	-1.101123923	0.012013366
YPEL3	-1.130612105	0.012098064
RP11-436H11.1	-1.011743718	0.012205816
SNHG16	-1.043417365	0.012709169
MRPS21P1	-1.007164311	0.012740468
CKB	-1.003410717	0.012809479
AC079834.1	-1.389512158	0.012896315
TTC22	-1.009309707	0.013056241
RPL23AP65	-1.152846183	0.013105802
MT-TF	-1.306000988	0.01333595
RPS10	-1.283419998	0.013494824
CTD-2666L21.3	-1.19666038	0.013557876
NOP56P1	-1.158656907	0.013628415
RP11-397P13.7	-1.105896201	0.013690813
AP001350.4	-1.164761764	0.013756479
RP11-467I20.3	-1.110635112	0.013820261
RP5-1092A3.4	-1.243468447	0.014013671

RPL10P3	-1.28734515	0.014090975
RPL30P14	-1.343503688	0.014208449
RP3-399J4.2	-1.171359704	0.014248195
RPS24P17	-1.111108853	0.014258644
RP11-428G5.1	-1.281284308	0.014348851
AC007969.5	-1.227226841	0.014400048
VAMP5	-1.146623799	0.014423147
RP11-478C6.4	-1.415819638	0.014579143
RPL27A	-1.047606395	0.014622353
RP11-83N9.5	-1.070987119	0.014631641
RP1-130G2.1	-1.190267946	0.014816927
RPS18P12	-1.007981288	0.014834991
TBX15	-1.300859585	0.01487454
TMEM191B	-1.053304665	0.014882367
RP11-832N8.1	-1.074019913	0.014942713
RPS15	-1.110631903	0.014963262
CTC-484M2.1	-1.148117905	0.014974278
RP11-621K7.1	-1.073403353	0.015036288
RP11-174N3.4	-1.106956167	0.015376069
UCN	-1.334753366	0.015718066
RPL13AP7	-1.322454389	0.01587758
LYPD6B	-1.157269808	0.015917191
RP11-182J1.3	-1.30881428	0.016077935
RPPH1	-1.322454389	0.01649898
RP4-630J13.1	-1.117187528	0.016522441
HIST2H2AA3	-2.144661417	0.016555157
RP1-43E13.2	-1.121820861	0.016896823
RP11-3L10.3	-1.175988798	0.017040815
PHLDA1	-1.953303643	0.017343001
RP11-68I3.11	-2.403717743	0.017392437
PPP1R32	-1.481484812	0.01753998
AC006445.6	-1.105927651	0.01760922
AC005262.4	-1.301418751	0.017661617
RP11-477I4.4	-1.561930151	0.017964116
AC016708.2	-1.06503443	0.01801303
RP11-366M4.17	-1.203831856	0.018025273
KANSL1-AS1	-1.257445089	0.01805378
OST4	-1.076567714	0.018208225
PMS2P5	-1.044579948	0.018648876
RPL38P3	-1.142280641	0.018743821
AC091133.1	-1.048631359	0.018876649
TMSB10P1	-1.024237705	0.018967964
PIK3CD-AS2	-1.042432808	0.019305368
RP5-827C21.4	-1.365763503	0.019513925
ZNF436-AS1	-1.082254543	0.019948345

SNORD3B-2	-1.372925636	0.019974194
LYG1	-1.296179041	0.020364673
OTUB2	-1.105506125	0.020386194
RP4-570O12.2	-1.000727659	0.020657865
RP11-12M9.3	-1.022033994	0.020815616
SNHG19	-1.355407286	0.0208634
ZSCAN16-AS1	-1.162977156	0.021150217
C19orf53	-1.020527119	0.021193591
HIST1H2BJ	-1.602804835	0.021343788
AC007365.4	-1.170650044	0.021714442
DUSP2	-1.23970969	0.022512751
RRAS	-1.009372984	0.022629812
EML2-AS1	-1.13877937	0.022759968
RPS20P5	-1.075818933	0.023859271
RPS17	-1.023656271	0.023981911
RPL32P9	-1.056181932	0.023989954
RP11-336K24.12	-1.08503141	0.024093218
HSPB1P1	-3.213344349	0.02419316
CTC-543D15.8	-1.025850867	0.025151591
RP11-380G5.3	-1.104819658	0.025301898
FAM89B	-1.216393591	0.025785122
CCR7	-1.826453125	0.025830195
RP11-410L14.2	-1.053305676	0.025970909
CTC-338M12.5	-1.193531421	0.026449727
SNORD3A	-2.709890694	0.026519119
RP1-72A23.3	-1.118701056	0.027212492
CTD-2583A14.11	-1.153613783	0.027283975
C6orf3	-1.787266205	0.027776839
RPL13AP3	-1.080144413	0.028450638
AL353644.10	-1.017489388	0.0288996
RP11-16E12.1	-1.481687787	0.028930867
NRARP	-1.576166272	0.029009802
SELPLG	-1.038299223	0.029305122
RP5-1052M9.1	-1.211030375	0.029556042
HSPB1	-3.317316128	0.02961834
TMEM191A	-1.427361128	0.029990674
RP11-1348G14.1	-1.866956892	0.030124842
HIST2H2BF	-2.08821601	0.030262988
CTC-524C5.2	-1.452658857	0.031187375
RP11-225H22.7	-1.244869816	0.031349328
RP3-395M20.12	-1.064683839	0.031453423
CTA-351J1.1	-1.123262062	0.031843006
RPL23AP31	-1.354748511	0.032293665
MPZ	-1.13492862	0.032731443
MTND1P23	-1.069944204	0.033633705

FP671120.4	-1.013047267	0.03371521
RPS20P2	-1.103478443	0.034054764
AC005336.5	-1.087047526	0.034226331
RPL7P7	-1.241227181	0.035113425
C19orf24	-1.066089921	0.035998591
GPX1	-1.073116376	0.036074226
VKORC1	-1.046092253	0.036558752
MT-TQ	-1.025650866	0.037187332
AC008753.3	-1.115480004	0.037190515
SMIM24	-1.05263838	0.038153114
HES7	-1.563868881	0.038268346
CTSV	-1.004850048	0.040062869
TMEM86B	-1.015795828	0.040080155
S100A4	-1.101135672	0.04032462
LA16c-329F2.2	-2.025665499	0.040653264
AC079150.2	-1.194294797	0.041249366
SLC11A1	-1.425679827	0.042753154
IPO4	-1.19753012	0.04369839
RP11-359B20.1	-1.076869717	0.04443602
RP11-274B21.9	-1.068775308	0.044960821
DPM3	-1.102286619	0.04516499
NUDT8	-1.03026847	0.045907213
RP11-386G11.10	-1.067626725	0.046832027
SARNP	-1.074695654	0.046973455
TNFRSF10D	-1.028868059	0.047095443
JUND	-1.085948111	0.048497067
RP11-543P15.1	-1.267987203	0.048610142
AC139100.4	-1.12068496	0.048786201
TAGLN	-1.018217331	0.049208116
MAP6D1	-1.000564708	0.049691251
RP11-694I15.7	-1.122185424	0.049703857

Translationally up-regulated genes

Gene Name	Log Fold Change	p-value
HNRNPDLP2	1.719622204	5.39E-05
CTD-2576D5.2	1.489249288	0.000164399
NOL4	1.710590207	0.000384959
CTC-459F4.9	2.156150386	0.000392732
RP11-344P13.3	1.652635	0.000393729
MT-TT	1.399598784	0.000395966
RP11-232D9.1	1.439907151	0.000417043
IMMP1LP1	2.112829451	0.000541277
RP11-553K23.2	2.556101695	0.000563704
MTATP8P1	1.871164508	0.000754916

RP4-569D19.5	1.586331574	0.000869894
ACVR1	1.395137648	0.00104338
CTC-303L1.1	1.791954957	0.001108697
MAP10	1.170897814	0.001147472
CTC-281F24.1	1.071424354	0.001450557
GHET1	1.185743978	0.001761895
PAPOLB	2.155334382	0.001778022
LACC1	1.08833044	0.002251375
RP11-673E1.3	1.698690377	0.002282957
GEMIN2P1	1.042670259	0.002923329
RP11-105N14.1	1.46864935	0.003030174
TSEN15P1	1.691682838	0.003064458
BX322557.10	1.279975007	0.003097402
PTPN13	1.274698028	0.003168919
ZC3HAV1L	1.733469932	0.003189413
TEX9	1.056193616	0.003219444
DNAJC3-AS1	1.08419342	0.003281168
RP4-814D15.1	1.343184403	0.003534631
PKD1P5	1.137985403	0.003565533
C5orf34	1.061437204	0.004254642
RP11-467L13.7	1.361330992	0.004554833
CCDC18	1.131170011	0.004607245
CENPI	1.034345696	0.004693323
CTC-523E23.3	1.034139717	0.004714193
LRRCC1	1.433049661	0.004904936
CCDC141	1.178668682	0.004910831
SRSF9P1	1.060327281	0.004938248
CDKL5	1.244147305	0.005297763
ANKRD20A17P	1.829561343	0.005306283
GS1-124K5.3	1.325512055	0.005382958
HAUS1P3	1.354859052	0.005439124
TPMTP1	2.067095616	0.006091992
CLECL1	1.085162973	0.00610045
ATG4C	1.197037675	0.006392016
MIR570	1.24218712	0.006427174
NSRP1P1	1.027976801	0.006442889
AC000068.5	1.271882549	0.007195101
RP11-958N24.1	1.461161632	0.00725238
KRIT1	1.209573268	0.007393916
PCDHGA1	1.30413416	0.007410392
HERC2P3	1.24090065	0.007463981
SLC35G2	1.257835031	0.008007398
DHFRP1	1.326847927	0.008424886
MPRIPP1	1.188081291	0.008760808
EIF2S2P3	1.194178962	0.008962068

AC007395.4	1.298942898	0.009494797
MRPL30P1	1.105003772	0.009832898
ZMAT1	1.214874067	0.00991755
MRPS10P1	2.22004447	0.009979205
JAK2	1.098948962	0.010044798
FAM135A	1.252647679	0.010069257
CETN3	1.07994577	0.010118309
RP11-332L8.1	1.169992165	0.011367238
ZNF85	1.172609418	0.011749004
ZNF534	1.131145447	0.011884447
PCMTD2	1.013010615	0.012291321
RP11-255H23.2	1.045222089	0.012299203
VDAC1P5	1.023722965	0.012306256
AC142528.1	1.469505337	0.012333254
HMGN2P46	1.299669977	0.012346341
TERF1P5	1.253869116	0.012607351
YEATS4	1.189904212	0.012612395
IGLV4-60	1.229597633	0.012867261
RP11-451O18.1	1.140303973	0.013141341
NUDT7	1.002910747	0.013484247
RP13-140E4.1	1.2910199	0.013554627
HNRNPA1P70	1.001564711	0.013662564
RP11-517P14.2	1.397462702	0.013884525
SLC25A20P1	1.064918597	0.014050777
SLC35A3	1.074806144	0.014078788
SMN1	1.017198295	0.014191805
GPN3	1.02395985	0.014596527
LYPLAL1	1.165123933	0.014618127
RELL1	1.149079307	0.014743426
GPR180	1.064275106	0.01509458
RP11-397E7.2	1.46906111	0.015825137
SPAG1	1.151824357	0.015842938
ZNF860	1.313187661	0.016386718
FAM72C	1.136166512	0.016405624
RP11-314A15.2	1.250283705	0.01650315
ANKRD26	1.014040187	0.016659859
CTD-2561J22.2	1.32196896	0.016715561
RP11-220H4.6	1.288980323	0.016927194
TET1	1.09620144	0.017145519
GATSL2	1.279707122	0.017516199
RP11-95M15.2	1.745573436	0.018240747
RP11-817I4.1	1.05794676	0.018631238
RP4-595K12.2	1.220623296	0.018682096
DUSP6	1.068028707	0.019162937
RP11-488L18.4	1.097328017	0.019256624

RP11-1319K7.1	1.102603338	0.019455901
ZNF711	1.248538544	0.019482619
GMCL1P1	1.623479828	0.019663254
TRAM1L1	1.314195535	0.019744863
AF013593.1	1.266479203	0.019859787
RP11-51C14.1	1.253497063	0.020116796
HAUS1P2	1.093542648	0.020207574
EEF1E1	1.082172197	0.020265147
CNOT6LP1	1.875279891	0.020322573
HTATSF1P2	1.664865028	0.020410093
ADAM9	1.130425915	0.020458973
RP11-142L4.2	1.414217574	0.020686152
RP11-397J20.1	1.11997118	0.020708516
RP11-1070N10.5	1.09699749	0.021185733
HPRT1	1.164210323	0.021378408
CCNE2	1.033431308	0.021783957
RP11-27I1.4	1.07352001	0.022018895
ENTPD1-AS1	1.460050799	0.022172038
CD84	1.083066992	0.022441114
RP11-320A16.1	1.571901468	0.022917351
ERBB4	2.046691107	0.022995145
AC097523.3	1.16613671	0.023259613
AC008592.3	1.209264845	0.023413038
REL	1.115033712	0.023488215
C9orf129	1.449771279	0.02412269
KRT8P46	1.527646896	0.02456208
UBASH3B	1.043941239	0.024777119
SLC25A15	1.129880289	0.025077921
ZMYM1	1.076933995	0.025086951
CTAGE14P	1.000507156	0.02511076
RSL24D1P11	1.327467991	0.025257665
RP11-629B11.4	1.098849805	0.025419257
SCN8A	1.010593312	0.02594153
CMTM8	1.083127567	0.026085753
RP1-179N16.3	1.062729743	0.026233212
RP11-142E9.1	1.08688411	0.02695308
ZNF724P	1.660314961	0.027070231
PTP4A1P7	1.395137648	0.027653568
RAD51AP1	1.140051842	0.027672868
PAICSP5	1.050259885	0.028309835
RP11-288C17.1	1.072369454	0.028318189
ZNF92	1.220072364	0.02885777
RP11-385M4.3	1.181847834	0.029229078
RGS18	1.282933417	0.029798764
RP11-553L6.5	1.536512778	0.029822941

RP11-498D10.8	1.074151972	0.030275838
RP3-510O8.3	2.349940138	0.030276773
TMEM9B-AS1	1.061535953	0.03055203
RP11-621H8.2	1.16111643	0.030639047
ANAPC1P1	1.174910301	0.030836229
FAM161A	1.200731835	0.031276781
ZNF680	1.197899034	0.03135027
RP11-134K13.2	1.061712521	0.032221793
HPGD	1.408954078	0.032280555
SNX24	1.056428914	0.032683795
RP11-3J10.7	1.040545133	0.032714464
RP3-468K18.6	1.690947973	0.032795045
RP11-68L18.1	1.101621128	0.032893995
KB-1507C5.4	1.21982789	0.033078708
CTA-246H3.11	1.12913471	0.033293549
FDX1P1	1.177076393	0.033536721
RP11-181C21.4	1.350971408	0.033847505
ATRNL1	1.779956005	0.034005228
CTB-133G6.1	1.601657275	0.034804737
BCAS2P2	1.460687833	0.0349514
CNTF	1.597694593	0.034956689
RP11-307P22.1	1.108789134	0.034982391
EML5	1.240658841	0.035051673
AP1AR	1.041291093	0.035145855
XPOTP1	1.040856896	0.035201745
RAD51AP1P1	1.496679207	0.035270654
FBXO4	1.014008222	0.036893108
RP11-5N11.1	1.057717111	0.03708167
STK3	1.067926712	0.03720401
PPATP1	1.247472093	0.038591566
LRRFIP1P1	1.202874919	0.038759312
DTX2P1	1.054104584	0.038901011
RP11-159G9.5	1.64842843	0.039345467
AC005780.1	1.254501546	0.039497268
RMI1	1.155232239	0.041024661
AC078899.1	1.577356198	0.041093032
CDC27P1	1.046774234	0.041342772
RP11-360F5.3	1.060280708	0.041503203
C3orf20	1.025992391	0.041910217
CTD-2144E22.10	1.072059949	0.043371202
CTD-2081C10.7	1.766332281	0.043738249
HAUS6P1	1.933850334	0.044764258
ERC2	1.098281458	0.045463091
RP11-700P18.2	1.504951791	0.045659239
LRRC37A15P	1.292201529	0.045786602

AC005154.7	1.054329392	0.046324492
RP11-206L10.2	1.683001575	0.047349805
AC092646.2	1.319255045	0.047431809
RP11-114M5.1	2.141296689	0.047681754
RAD1P1	1.251333093	0.049446538
RP11-553D4.2	1.137368862	0.049935331

Transcriptionally and translationally regulated genes

Gene Name	Log Fold Change	p-value
HIST1H2AE	-2.538934092	0.005553168
AC062017.1	-2.142306014	5.78E-05
RP11-642A1.2	-2.072667318	0.010875327
ASB12	-1.479696944	0.001847889
RBMS2	-1.452977315	0.010727903
HIST2H2BE	-1.416516194	0.034787543
CD55	-1.370213667	0.019293418
TNNT3	-1.258271938	0.006409854
APOE	-1.21648618	0.018909913
FSCN1	-1.140083757	0.018309068
TMEM71	-1.123468716	0.015831723
DLX2	-1.016939402	0.015880936
LINC01252	1.327795227	0.011147186

Supplemental Table 2

<u>Negative Regulators of Apoptosis (translationally downregulated):</u>

TNF receptor superfamily member 10d (TNFRSF10D)
Adipogenesis associated Mth938 domain containing (AAMDC)
Heat shock protein 90 beta family member 1 (HSP90B1)
Heat shock protein family B (small) member 1 (HSPB1)
Msh homeobox 1 (MSX1)
Myelin protein zero (MPZ)
Nerve growth factor receptor (NGFR)
Sequestosome 1 (SQSTM1)
Urocortin (UCN)
Vascular endothelial growth factor B (VEGFB)
